# Histone Methylation Restrains the Expression of Subtype-Specific Genes during Terminal Neuronal Differentiation in *Caenorhabditis elegans*

**DOI:** 10.1371/journal.pgen.1004017

**Published:** 2013-12-12

**Authors:** Chaogu Zheng, Siavash Karimzadegan, Victor Chiang, Martin Chalfie

**Affiliations:** Department of Biological Sciences, Columbia University, New York, New York, United States of America; University of Cambridge, United Kingdom

## Abstract

Although epigenetic control of stem cell fate choice is well established, little is known about epigenetic regulation of terminal neuronal differentiation. We found that some differences among the subtypes of *Caenorhabditis elegans* VC neurons, particularly the expression of the transcription factor gene *unc-4*, require histone modification, most likely H3K9 methylation. An EGF signal from the vulva alleviated the epigenetic repression of *unc-4* in vulval VC neurons but not the more distant nonvulval VC cells, which kept *unc-4* silenced. Loss of the H3K9 methyltransferase MET-2 or H3K9me2/3 binding proteins HPL-2 and LIN-61 or a novel chromodomain protein CEC-3 caused ectopic *unc-4* expression in all VC neurons. Downstream of the EGF signaling in vulval VC neurons, the transcription factor LIN-11 and histone demethylases removed the suppressive histone marks and derepressed *unc-4*. Behaviorally, expression of UNC-4 in all the VC neurons caused an imbalance in the egg-laying circuit. Thus, epigenetic mechanisms help establish subtype-specific gene expression, which are needed for optimal activity of a neural circuit.

## Author Summary

As neurons differentiate they express specific genes that give them their distinctive shapes, activities, and functions. Much of this differentiation is controlled by the expression of transcription factors, proteins that turn on the expression of other genes. We find, however, that another aspect of terminal neuronal differentiation is the removal of inhibitory constraints on gene expression. These constraints often involve the modification of DNA or of general DNA binding proteins such as histones. This modification, referred to as epigenetic regulation, can activate or inactive genes without changing the genetic material. We found that the differentiation of nematode motor neurons was affected by genes involved in histone modification. Specifically, a gene that is needed in a subset of the motor neurons is initially turned off in all cells by histone modification. Mutation of histone modification genes causes the gene to be on in all cells. Normally, however, this removal of the inhibition is triggered by an external signal that only affects the specific cells.

## Introduction

Epigenetic regulation of gene expression, e.g., through histone modification, is essential to silence key developmental genes, prevent neural differentiation, and maintain the pluripotency of embryonic stem cells (ESCs) [1]. For example, methylation on the lysine 27 of histone 3 (H3K27) suppresses the expression of genes required for the neural lineage and prevents the differentiation of the mammalian ESCs into neural precursor cells (NPCs); ESCs deficient in the Polycomb-group (PcG) proteins, which promote H3K27 trimethylation, show an increased propensity to differentiate [2], [3]. H3K27 trimethylation and histone deacetylaiton are also responsible for silencing neuron-specific genes and inhibiting neurogenesis during the differentiation of NPCs into astroglial cells [4]. Despite the importance of epigenetic control in the cell fate choice of stem cells or neural precursor cells, the involvement of chromatin modification in the terminal differentiation of neurons has not been reported.

Here we show that histone methylation restrains the expression of a functionally important transcription factor (TF) in a specific neuronal subtype in *Caenorhabditis elegans*. Within the ventral cord of *C. elegans* the six VC motor neurons help control egg laying [5]. VC neurons can be categorized into two subtypes according to their proximity to the vulva, their morphology, and their gene expression. The vulva VC neurons, VC4 and VC5, flank the vulva, have short processes in the ventral cord, and send branched processes dorsally along the vulval hypodermis on each side of the vulval slit. In contrast, the nonvulval VC neurons, VC1-3 and VC6, which are more distant from the vulva, send less-branched processes to the vulva and have longer processes in the ventral cord. All VC axons extend dorsal branches that innervate vm2 vulval muscles, but only VC1-3 and VC6 innervate ventral body muscles. All VC neurons generate acetylcholine (ACh), but its activity is only known for the vulval VC cells where it acts as a neuromodulator that inhibits the activity of egg-laying-inducing HSN motor neuron [5]. In addition, only the vulval VC cells release serotonin to activate vulval muscle and promote egg laying [5]. Since loss of VC4 and VC5 neurons increases egg laying [6], their overall activity is biased toward inhibition.

We find that the vulval VC neurons, but not the nonvulval VC neurons, express the TF UNC-4 and that this expression is determined by both external signals from the vulva, which trigger *unc-4* transcription in the adjacent vulval VC neurons through EGF signaling, and internal histone methylation, which silences *unc-4* in the nonvulval VC neurons in the absence of EGF signals. Mutation of the H3K9 methyltransferase MET-2, the human HP1 homolog HPL-2 and the MBT repeats-containing protein LIN-61, which are recruited to H3K9me2/3, and a novel chromodomain protein CEC-3 leads to the loss of subtype-specificity of *unc-4* expression; the gene is expressed in all six VC neurons. Epigenetic silencing of *unc-4* occurs initially in all six neurons, but is relieved in the vulval VC cells due to the action of EGF signaling and the LIM domain TF LIN-11. Functionally, this release of epigenetic silencing of *unc-4* expression in the vulval VC neurons helps balance the choice between egg retention and egg laying.

## Results

### Only the vulval VC neurons express *unc-4*

The transiently expressed UNC-4 homeodomain protein plays an important role in the differentiation and synaptic formation of ventral nerve cord motor neurons in *C. elegans* [7]. To monitor the dynamics of *unc-4* expression pattern, we used a 2.5 kb promoter of the *unc-4* gene (*unc-4p*) to drive the expression of a rapidly degraded form of GFP (*uIs45*; [8]). When compared to the transgene of *unc-4* promoter-driven regular GFP, the expression from *uIs45* labeled far fewer cells at nearly every developmental stage (Figure S1). *uIs45* expression began in DA neurons in 3-fold embryos and lasted until the middle of first larval (L1) stage. The reporter was expressed next in the VA neurons beginning with the most anterior cells during the L2 stage. This expression was lost soon afterward; by the late L2 stage the most posterior VA neurons had expressed and then lost the reporter (Figure S2). Although head neurons SAB, AVF, and I5 constantly expressed the reporter throughout the larval and adult stages, virtually no ventral cord neurons expressed it from the L3 to early L4 stage.

**Figure 1:**
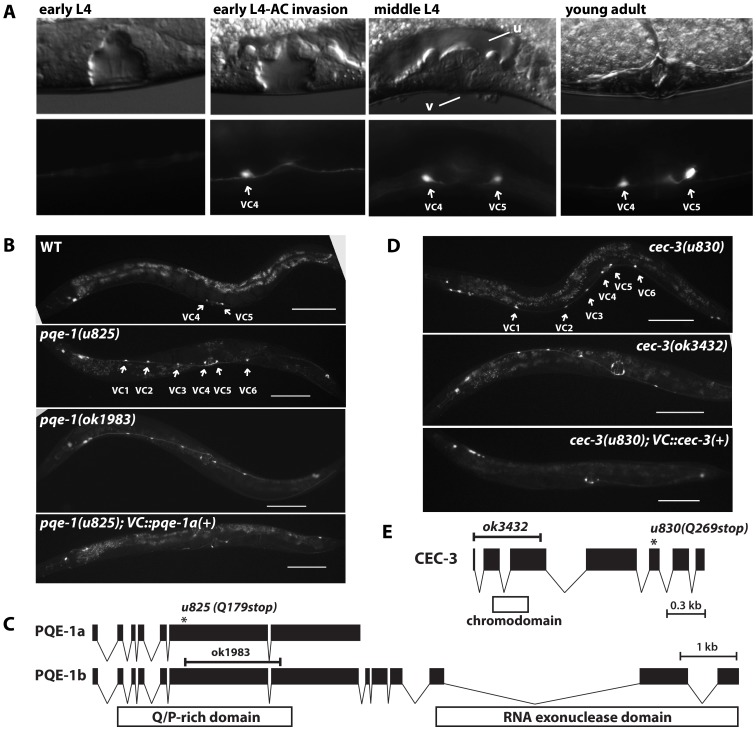
Mutation of *pqe-1* and *cec-3* leads to ectopic expression of *unc-4* in all VC neurons. (A) Expression of MDM2::GFP from *uIs45[unc-4p::MDM2::GFP]* in VC4 and VC5 neurons at various stages of vulval developmental. The lower image represents signals from the GFP channel, while the upper image is a DIC image. Letter “v” and “u” represents vulva and uterus. (B) Expression of *uIs45* in adults from wild type, *pqe-1(u825)*, *pqe-1(ok1983)*, and *pqe-1(u825)* with *pqe-1a* isoform driven by a VC-specific promoter. Scale bar = 100 µm. (C) The structure of the *pqe-1* gene, the position of predicted domains, and the positions of the *u825* and *ok1983* mutations. (D) Expression of *uIs45* in adults of two different *cec-3* mutants and *cec-3(u830); uIs45* animals with *cec-3(+)* driven by a VC-specific promoter. Scale bar = 100 µm. (E) The structure of *cec-3* gene, the position of chromodomain, and the positions of the *u830* and *ok3432* mutations.

The reporter was expressed in VC4 and VC5 (the vulval VC neurons; Figure 1A) beginning at the same time as anchor cell invasion in early L4 animals. The expression stabilized in the mid-L4 when the hermaphrodite vulva formed (Figure 1A) and lasted throughout adulthood. The reporter was not observed in the other VC neurons at any time. In males, no ventral cord neuron expresses the reporter after L3 stage despite the expression in VA and DA neurons during earlier larval stages.

### Genetic screen identified mutants with abnormal *unc-4* expression pattern

We screened F2 animals representing 25,000 haploid genomes after EMS mutagenesis for mutants with increased expression of *uIs45* in adult ventral cord neurons. Twenty-three mutants had more than the two neurons found in the parent strain (Table S1). Twelve mutants with strong phenotypes were identified by whole genome sequencing (see Methods) and had defects in three genes (*pqe-1*, *cec-3*, and *ceh-20*). The remaining eleven mutants have either weak phenotypes or low penetrance and were not studied further; all complemented null alleles of *pqe-1*, *cec-3*, and *ceh-20*.

Of the twelve mutants we analyzed, the eight *pqe-1* mutants and the two *cec-3* mutants expressed *uIs45* in all six VC neurons, whereas the two *ceh-20* mutants prolonged *uIs45* expression in adult VA neurons (Figure S3). In this paper we focus on the abnormal activation of *unc-4* promoter in the VC neurons and the mechanisms inhibiting *unc-4* expression in these neurons.

### Mutation of *pqe-1* results in ectopic *unc-4* promoter expression in all six VC neurons

*pqe-1* was originally identified as a modifier of polyglutamine neurotoxicity; mutation of *pqe-1* significantly enhanced polyglutamine-induced neurodegeneration of the ASH neurons [9]. All eight alleles in our screen harbored nonsense mutations and caused *uIs45* expression in all six adult VC neurons with 100% penetrance (Table S1; Figure 1B). The allele *u825* was used in subsequent studies. The extra GFP-expressing cells in these animals were identified as the VC1-3 and VC6 neurons because the labeled cells were in the correct anatomical positions and hermaphrodite-specific. Furthermore, the expression of a RFP version of *uIs45* overlapped with the expression of the VC marker *vsIs13[lin-11p::pes-10p::GFP]* [6] in these extra cells, confirming their identity (Figure S4).

The *pqe-1* gene encodes two isoforms: the a isoform has a glutamine/proline-rich domain, whereas the b isoform has an additional C-terminal RNA exonuclease domain ([9]; Figure 1C). Because, *ok1983*, an allele that deletes a large portion of both isoforms and causes a subsequent frame shift, produced the same ectopic *uIs45* expression as our eight *pqe-1* alleles, all these alleles are likely to be null alleles. Additionally, since expression of the *pqe-1a* isoform from the VC-expressed promoter *lin-11p::pes-10p*, which contains a 500 bp *lin-11* enhancer and a *pes-10* basal promoter [6], prevented the ectopic *unc-4* expression, PQE-1 acts cell-autonomously and does not require the exonuclease domain (Figure 1B). Consistent with cell-autonomous activity, 4.7 kb of DNA upstream of the start codon of *pqe-1* drove RFP expression in ventral cord neurons including the VC neurons (Figure S5) and in head and tail neurons. Because *lin-11p::pes-10p* is also expressed in 2° vulval cells, uterine pi cell progeny, and the spermatheca, we repeated the rescue experiments by expressing *pqe-1a*(+) from the *ida-1* promoter [10], which is expressed in many neurons but only overlaps with the expression pattern of *lin-11p::pes-10p* in the VC cells. We obtained similar results (data not shown). In this paper we show results of VC cell-specific rescue experiments with the *lin-11p::pes-10p* promoter, but all of the results were confirmed with *ida-1* promoter-driven transgenes. Moreover, to rule out the possible non-cell autonomous interactions among the VC neurons, which synapses on one another [11], we also performed mosaic analyses on *pqe-1(u825); uIs45* animals with an extragenic *pqe-1(+)* array (see Text S1 for the method). In the 15 mosaic animals we examined, all VC1-3 and VC6 cells lacking the rescuing array expressed GFP strongly, suggesting that *pqe-1* acts cell autonomously.

### Chromodomain proteins and histone methyltransferases also restrict *unc-4* VC expression

The nonsense mutation *cec-3(u830)* produced the same phenotype as the *pqe-1* mutations. *cec-3* encodes a chromodomain-containing protein. The *ok3432* allele, which deletes the start codon of CEC-3 gave the same ectopic expression of *uIs45* in all adult VC neurons (Figure 1D and 1E) and failed to complement *u830*, suggesting that *u830* is also a null allele. Moreover, CEC-3 expression in VC neurons restored the normal *unc-4* promoter expression pattern, indicating that it acts cell-autonomously in the VC neurons (Figure 1D). As with *pqe-1*, mosaic analysis confirmed the *cec-3* cell autonomy in individual VC cells (data not shown).

**Figure 2:**
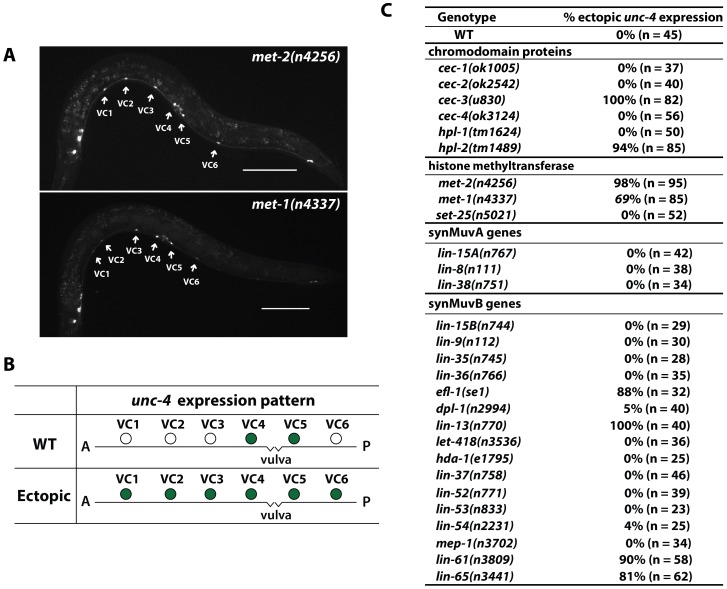
Ectopic expression of *unc-4* in all six VC neurons in animals with mutations affecting chromodomain proteins and histone methyltransferases. (A) Expression of *uIs45* in *met-2(n4256)* and *met-1(n4337)* adults. Scale bar = 100 µm. (B) Illustration of the wild-type and ectopic *unc-4* expression pattern in adult animals. Filled circles represent cells that express *uIs45*. (C) The penetrance of the phenotype of *unc-4* ectopic expression in various mutants.

Because the CEC-3 protein has a chromodomain, a domain which binds to repressive histone modifications and generally mediates transcriptional suppression [12], we suspected that the abnormal expression of *uIs45* in *cec-3* mutants resulted from dysregulation of epigenetic silencing. Indeed, mutants defective in the histone H3K9 methyltransferase gene *met-2* [13] did express *uIs45* in all VC neurons (Figure 2A). Mutations in another histone methyltransferase MET-1, which mainly promotes H3K36 methylation [14] but affects the abundance of H3K9 methylation [13], also resulted in ectopic *unc-4* expression. The *met-2* animals showed higher penetrance and brighter GFP expression in VC1-3 and VC6 cells than the *met-1* mutants (Figure 2A and 2C), consistent with previous reports that MET-2 plays a major role in promoting H3K9 methylation, whereas MET-1 is a minor contributor [13].

MET-2 is the *C. elegans* homolog of human SETDB1 and Drosophila Eggless [15], which specifically trimethylate H3K9 and contribute to HP1-mediated silencing of euchromatic genes [16]. Andersen et al. [13] found that *met-2* mutant embryos had significantly less H3K9 trimethylation. Towbin *et al.* [17], however, subsequently showed that MET-2 was specific to mono- and di-methylation of H3K9, and another histone methyltransferase (HMT), SET-25, mediated H3K9 trimethylation in the germ line and embryos. We found that *set-25* mutants did not show ectopic *unc-4* expression (Figure 2C), suggesting H3K9 dimethylation may be mainly responsible for the repression of *unc-4* in VC1-3 and VC6. Because we cannot rule out the possibility that MET-2 or some other HMTs promote H3K9 trimethylation in adult VC cells to silence *unc-4*, we have designated the modification caused in the VC cells as H3K9me2/3.

Loss of MET-1 affects both H3K36 and H3K9 trimethylation in embryos [13] and either or both activities could be important for the effect on *unc-4* expression. If the action of MET-1 is direct, the latter activity is likely to be important for the repression of *unc-4*, since 1) H3K36me3 is an epigenetic mark present in the coding sequence of actively transcribed genes [18]; and 2) mutation of *mes-4*, another HMT responsible for at least germline and embryo H3K36me3 [19], did not result in ectopic *unc-4* expression (data not shown). A more indirect effect of MET-1, however, involving H3K36 trimethylation may also occur.

We also examined mutants defective in other chromodomain-containing proteins, which are thought to be tissue or gene specific [20], and found that mutation of *hpl-2* but not others genes in this family led to similar ectopic expression of *uIs45* (Figure 2B and 2C). HPL-2 is the *C. elegans* homolog of human HP1 and is known to be recruited to H3K9me2/3 [21], [22]. Therefore, this result suggests that HPL-2 and perhaps the other chromodomain protein CEC-3 mediate the transcriptional repression of *unc-4* through H3K9 methylation in VC1-3 and VC6 neurons. In addition, mutation of genes encoding the components of the Polycomb-like chromatin repressive complex (*mes-2*, *mes-3*, and *mes-6*), which promote H3K27 methylation, did not cause ectopic *unc-4* expression (n>50 for each mutant). Similarly, treatment with histone deacetylase inhibitors (valproic acid or Trichostatin A) had no effect on the expression pattern of *unc-4* (n>50 in both cases). Therefore, H3K9 methylation may contribute most to the silencing of *unc-4*.

### Mutation of *pqe-1*, *cec-3* and *met-2* elevates *unc-4* transcription in VC1-3 and VC6 neurons

**Figure 3:**
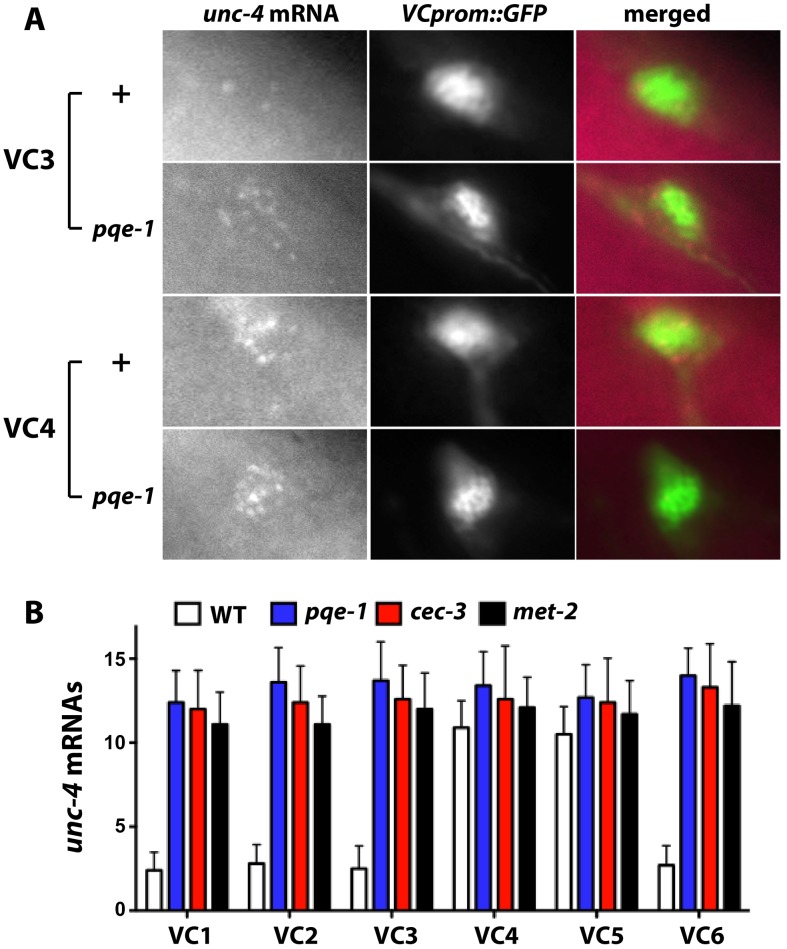
Endogenous *unc-4* transcripts accumulate in all six VC neurons in *pqe-1*, *cec-3*, and *met-2* mutants. (A) Fluorescently labeled *unc-4* mRNA is found in VC4 but not VC3 neurons in wild-type animals, but in both cells in *pqe-1* mutants. (B) Average number of fluorescently labeled *unc-4* mRNA transcripts in each VC neurons from wild-type, *pqe-1*, *cec-3*, and *met-2* animals (n≥20).

Yamada *et al.* reported that *pqe-1* mutations increased transgene expression but not endogenous gene expression [23]. In contrast, using single molecule fluorescence *in situ* hybridization, which individually labels at least 80% of the cellular mRNA [24], we found that the level of endogenous *unc-4* transcripts increased (Figure 3). VC1-3 and VC6 neurons in wild-type animals contained less than three fluorescently labeled *unc-4* mRNA molecules (VC1: 2.4±0.3, VC2: 2.8±0.4, VC3: 2.5±0.4, and VC6: 2.7±0.4; mean ± SEM, N = 20), whereas VC4 and VC5 had about 11 labeled *unc-4* transcripts (VC4: 10.9±0.5; VC5: 10.6±0.5). All VC neurons in *pqe-1*, *cec-3* and *met-2* mutants had >12 *unc-4* transcripts. These results confirmed that *unc-4* expression was significantly upregulated in VC1-3 and VC6 neurons in *pqe-1*, *cec-3*, and *met-2* mutants and that *uIs45* truly monitored endogenous *unc-4* promoter activity.

### Mutation of a subset of synMuv B genes also caused ectopic *unc-4* expression

In addition to regulating *unc-4* expression in the VC neurons, *hpl-2*, *met-1*, and *met-2* also repress transcription of *lin-3/EGF*, which induces vulval development [13], [25]. Because all three genes are synMuv B genes (mutation of any of them, together with a mutation in a synMuv A gene, leads to a synthetic multivulva phenotype [26]), we tested whether other genes regulating vulval development also controlled *unc-4* expression. Indeed, mutation of four other synMuv B genes, but no synMuv A genes, caused ectopic expression of *unc-4p::MDM2::GFP* in the VC neurons (Figure 2C). The four synMuv B genes were *efl-1/E2F*, which encodes a transcriptional repressor [27]; *lin-13*, which encodes a zinc-finger protein that forms a complex with HPL-2 and helps localize HPL-2 to certain genomic loci [28]; *lin-61*, which encodes a protein with four malignant brain tumor repeats that bind to di- and tri-methylated H3K9 [29], [30] and interacts genetically with *hpl-2* and *met-2* in vulva development [30]; and *lin-65*, which encodes a large acid-rich protein that lacks obvious similarity to non-nematode proteins [31].

*pqe-1* and *cec-3* were not, however, synMuv genes. Loss of either *pqe-1* or *cec-3* in the background of a class A or class B synMuv mutant, such as *lin-15A* or *lin-15B*, respectively, did not give a multivulva phenotype (data not shown). Thus, vulval and VC development utilize genetic pathways with overlapping yet divergent regulatory roles.

### *pqe-1* acts similarly to genes involved in epigenetic silencing

PQE-1 and proteins involved in chromatin modification and remodeling acted similarly in several different situations. *cec-3*, *met-2*, and *lin-13* mutations, like *pqe-1* mutations [23], enhanced transgene expression in AIZ neurons (Figure S6A), suggesting these genes act together to inhibit transcription. Similarly, loss of *cec-3*, *met-1*, *met-2* and *lin-13*, like loss of *pqe-1* [9], enhanced polyglutamine (polyQ) repeat-induced neurodegeneration in ASH neurons (Figure S6B; see Text S1 for the method). Moreover, Bates *et al.* found that mutation of several histone deacetylases increased this polyQ-dependent neurodegeneration [32], indicating that histone modification-induced transcriptional suppression was generally protective for polyQ-mediated neuronal cell death. The fact that PQE-1 shares similar functions with HMT and chromodomain protein hints that PQE-1 may also regulate chromatin silencing.

### The vulva-inducing signal triggers *unc-4* expression in VC neurons

The morphological differentiation of VC neurons requires guidance cues from vulval cells [33]. Only the vulva-flanking VC4 and VC5 neurons branch into the vulval region and innervated vulval muscle in wild-type animals. However, when the vulva is displaced anteriorly to lie between VC3 and VC4 in *dig-1* mutants, the axonal branching occurs in the now vulva-flanking VC3 and VC4 neurons, but not in VC5 [33].

**Figure 4:**
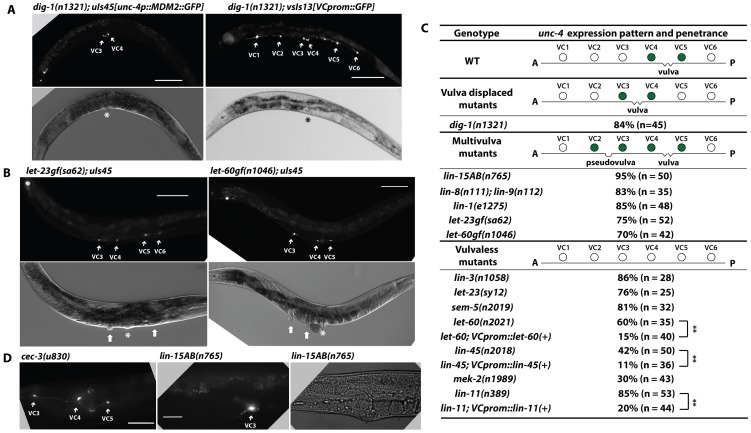
*unc-4* expression in the vulval VC neurons depends on signals from the developing vulva. (A) Expression of *uIs45* and VC marker *vsIs13* in *dig-1(n1321)* adults. The asterisk marks the position of the vulva. Scale bar = 100 µm. (B) *uIs45* expression in *let-60gf(n1046)* and *let-23gf(sa62)* multivulva adults. In the DIC images, the asterisk marks the real vulva, and thick white arrows point to pseudovulvae. (C) *uIs45* expression and penetrance in wild type and mutant animals. Filled circles represent cells that express *uIs45*. % defect denotes the percentage of total animals showing the defect. Double asterisks indicate that the two-tailed P value is less than 0.01 in a Fisher's exact test. (D) The morphology of VC neuron processes in *cec-3(u830); uIs45* and *lin-15AB(n765); uIs45* animals. The middle panel shows the branching of VC3 process into the pseudovulva in *lin-15AB(n765)* mutants. Scale bar = 20 µm.

*unc-4* expression in VC neurons also depended on similar external cues. In *dig-1* mutants, VC3 and VC4 neurons flanked the misplaced vulva and expressed *unc-4*, whereas VC5, which was no longer adjacent to the vulva, did not express *unc-4* (Figure 4A). Moreover, the positions of VC neurons were not changed in *dig-1* animals, and only VC3 and VC4 underwent morphological differentiation and migrated toward the vulva. These results indicate that the proximity to the vulva determines which VC neurons become the vulval subtype and activate *unc-4* transcription.

We also examined five Muv mutants, defective at various points in the pathway that controls vulval induction and development, and found they expressed *uIs45* in VC neurons flanking both the vulva and pseudovulvae (Figure 4B and 4C). Thus, the *unc-4* expression pattern in VC neurons is regulated by signals from vulval tissue. In addition, VC neurons flanking the pseudovulva, such as VC3 in *lin-15AB* animals, extended axons to ectopic vulval muscles, mimicking the normal differentiation of vulva-flanking VC4 and VC5 (Figure 4D). Since these morphological changes were not observed in ectopic VC neurons that expressed *unc-4* in *pqe-1* or *cec-3* or other mutant animals (Figure 4D), *unc-4* expression was not sufficient to induce these morphological changes. Moreover, both we and Bany *et al.* [6] found no defects in VC axonal processes in *unc-4* mutants, suggesting that *unc-4* was not needed for VC morphological differentiation. Other genetic pathways may control the axonal outgrowth of the vulval VC neurons. Therefore, the epigenetic regulation of *unc-4* does not determine all the aspects of VC subtype identity.

### EGFR signaling induces *unc-4* expression in vulval VC neurons

Since EGFR/RAS/MAPK signaling induces vulval development and differentiation [34], we tested whether mutation of genes in this pathway eliminated the expression of *unc-4* in VC4 and VC5 neurons. Indeed, animals defective in *lin-3/EGF* and *let-23/EGFR*, which are vulvaless, failed to express *uIs45* in VC4 and VC5 (Figure 4C and S7A). *let-60/RAS* and *lin-45/RAF* mutant, which lack downstream effectors of EGFR, also failed to express *unc-4* in vulval VC neurons (Figure 4C and S7B). Importantly, *unc-4* expression in VC4 and VC5 neurons was restored in animals with hypomorphic alleles of *let-60* and *lin-45* by VC-specific expression of the respective wild-type gene, indicating that the EGFR/RAS/RAF signaling cascade functions cell-autonomously in VC neurons (Figure 4C and S7C).

*unc-4* expression was also absent in VC neurons in mutants of *sem-5/GRB2* (encoding an adaptor protein that links RAS to EGFR; [35]) and *mek-2/MAPKK* (encoding the downstream kinase of RAS; Figure 4C; [36]). Using the VC marker *vsIs13*, we have confirmed the presence of the six VC neurons in all the mutants that have diminished *unc-4* expression in these cells (data not shown). These results further support the hypothesis that EGFR signaling is essential for inducing *unc-4* transcription in the VC4 and VC5 neurons. The expression of *unc-4* in the SAB, AVF, and I5 head neurons was not affected by mutations of the EGFR pathway, indicating that other mechanisms maintain the constant expression of *unc-4* in these neurons.

**Figure 5:**
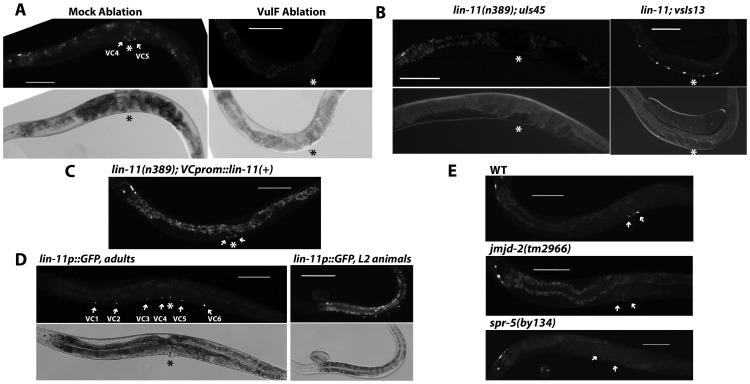
vulF cells, LIN-11, and histone demethylases are required to activate *unc-4* expression in vulval VC neurons. (A) Adult expression of *uIs45* in *syIs66[B0034.1::pes-10::GFP]* animals with vulF cells ablated at the L4 stage. The absence of *syIs66*, which labels vulF cells, confirmed their successful ablation. The asterisk indicates the position of the vulva. Scale bar = 100 µm. (B) Expression of *uIs45* and VC marker *vsIs13* in *lin-11(n389)* adults. (C) Effect of expression of *lin-11(+)* driven by an VC-specific promoter on *uIs45* expression in *lin-11(n389); uIs45* animals. The vulval VC neurons are indicated by arrows. (D) Expression of *syIs80[lin-11p::GFP]* in VC neurons in adults (left) and L2 larvae (right). (E) Adult expression of *uIs45* in wild-type, *jmjd-2(tm2966)*, and *spr-5(by134)* animals. Arrows indicate the positions of VC4 and VC5.

We next searched for the origin of the LIN-3/EGF signal that activates *unc-4* expression in VC neurons during vulval development. The two known sources of LIN-3 in vulval development are the anchor cell, which secretes LIN-3 at the middle to late L3 stage to induce primary vulval cell fate and pattern the vulval precursor cells [37] and the vulF cells, which secretes LIN-3 to signal the presumptive uv1 cells [38]. The anchor cell is likely not the source of LIN-3 for *unc-4* expression in the vulval VC neurons because *unc-4* expression occurred much later, after vulval precursor patterning was completed and the anchor cell invasion had started. Laser ablation of vulF cells caused the loss of *unc-4* expression in the VC4 and VC5 neurons (Figure 5A), suggesting that vulF cells are responsible for releasing EGF that activates *unc-4*. Moreover, the vulF cells, which are physically adjacent to the vulval VC neurons, are correctly positioned to activate *unc-4* expression in the latter cells with high concentrations of LIN-3/EGF. To further confirm the importance of vulF cells in inducing *unc-4*, we examined *lin-12d* mutants, in which all the six vulval precursor cells adopt the 2° vulval cell fate and the 1° lineage progeny vulF cells are not generated [39]. The strong *lin-12d* allele *n137* caused a multivulva phenotype but had no *unc-4* expression in any VC cells (N = 35 animals); the weaker *lin-12d* allele *n302* resulted in a vulvaless phenotype and the elimination of *unc-4* expression in VC4 and VC5 neurons (95% of 78 animals lacked expression in these cells). These results support the hypothesis that vulF cells are the source of the developmental signal that activates *unc-4*.

### *unc-4* expression requires LIN-11

We next wanted to identify the transcription factor (TF) that regulates *unc-4* expression in vulval VC cells. Although the ETS-domain-containing TF LIN-1 is a known nuclear target of the EGFR/RAS/MAPK signaling in vulval differentiation [40], loss of *lin-1* caused ectopic *unc-4* expression in nonvulval VC neurons and a multivulva phenotype instead of diminishing *unc-4* transcription (Figure 4C), suggesting that another TF may be involved in activating *unc-4* transcription. We screened six TFs (*egl-18*, *lin-11*, *tag-97*, *zag-1*, *vab-15*, and *hlh-3*) known to be expressed in VC neurons and found that mutation of *lin-11*, which encodes a LIM homeodomain protein [41], eliminated *uIs45* expression in VC4 and VC5 (Figure 5B). Expression of *lin-11(+)* using *lin-11p::pes-10p*, which is active in VC neurons but transiently expressed in the developing vulval cells, mainly the vulC and vulD cells but not the vulF cells [42], produced a normal *unc-4* expression pattern in *lin-11* mutants (Figure 5C). We obtained a similar rescue using the *ida-1* promoter, which is expressed in many neurons, including the VC neurons, but not vulval cells (data not shown). Thus, the action of *lin-11* on *unc-4* expression was cell autonomous.

*lin-11* expression in VC neurons started at the L2 stage (these cells are generated in the L1 stage) and all of the six VC neurons continued to express *lin-11* in subsequent larval and adult stages (Figure 5D). In contrast, *unc-4* expression in the vulval VC neurons began in the L4 stage and was absent in the nonvulval VC neurons, which also expressed LIN-11. Therefore, LIN-11 alone was not sufficient to activate *unc-4* transcription. Either LIN-11 induction of *unc-4* expression requires LIN-11 activation (e.g., through post-translational modification, other coactivators) or changes to downstream genes that allow it to act. Apparently upstream EGFR signaling is needed for these changes.

### Epigenetic silencing of *unc-4* is independent of the inductive EGFR pathway

**Figure 6:**
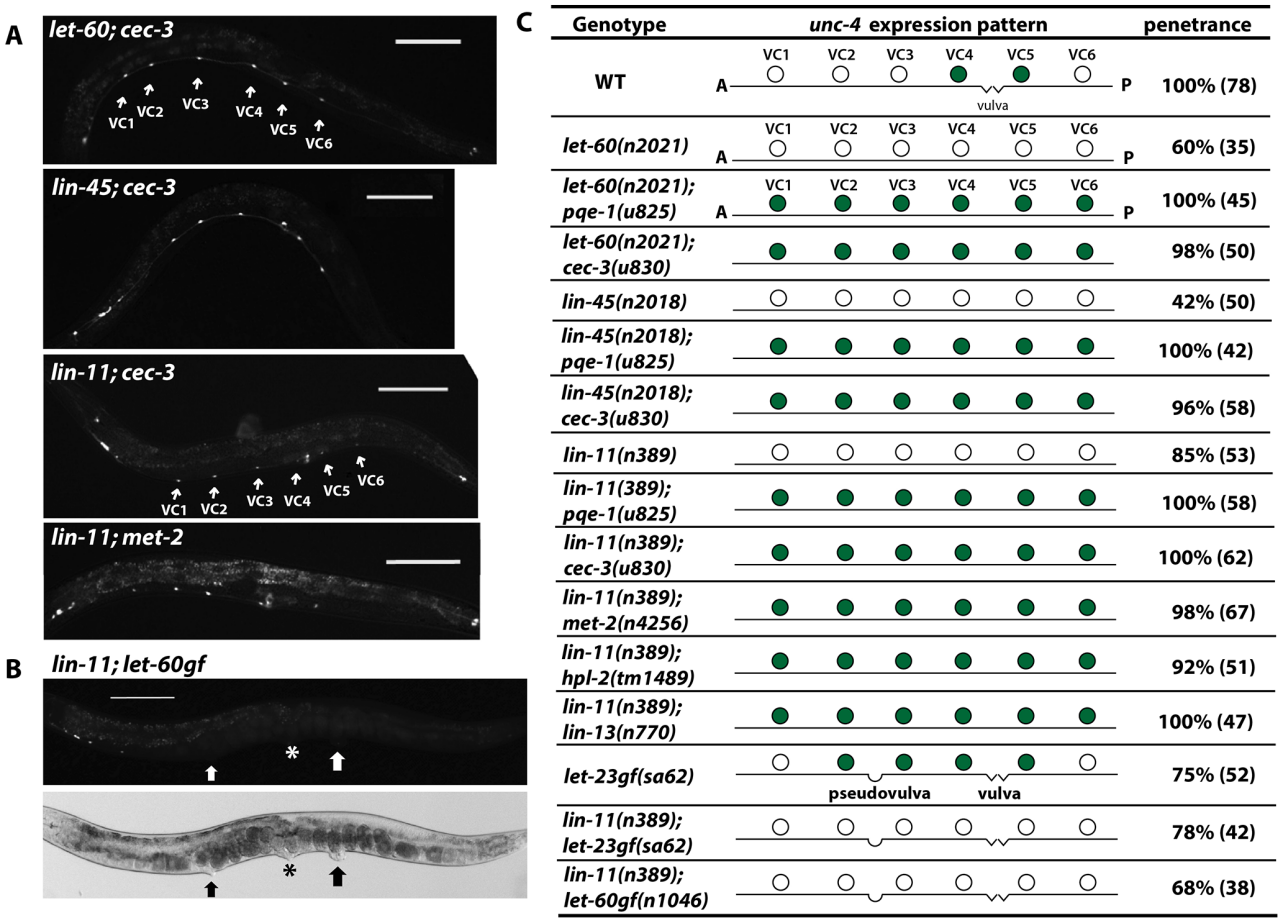
Epigenetic silencing of *unc-4* is independent of the inductive EGFR pathway. (A) Expression of *uIs45* in *let-60(n2021); cec-3(u830)*, *lin-45(n2018); cec-3(u830)*, *lin-11(n389); cec-3(u830)*, and *lin-11(n389); met-2(n4256)* double mutants. (B) Expression of *uIs45* in *lin-11(n389); let-60gf(n1046)* double mutants. The asterisk marks the real vulva, and thick arrows point to pseudovulvae. (C) *unc-4* expression and penetrance in various mutants. Filled circles represent cells that express *uIs45*.

Because genes affecting EGFR signaling activate *unc-4* expression and epigenetic factors maintain *unc-4* silencing in non-vulval VC neurons, we examined the relationship between these two categories of genes. *pqe-1* and *cec-3* were epistatic to *let-60*/RAS and *lin-45*/RAF (Figures 6), since all six VC neurons expressed *unc-4* in double mutants. These results suggest that the epigenetic factors that suppress *unc-4* expression are independent of the EGFR signaling that induces *unc-4* transcription. Importantly, the vulval neurons VC4 and VC5 lacking the epigenetic proteins still expressed *unc-4* in the absence of the inductive EGFR signaling, indicating that all six VC neurons expressed *unc-4* by default once the silencing mechanism was removed. These results also suggest that the EGFR signaling in vulval VC neurons overrides epigenetic silencing. Moreover, we noticed that the *unc-4* reporter expression was clear and strong from early L4 stage in *pqe-1*, *cec-3*, or *met-2* mutants, whereas expression in vulval VC cells of wild-type animals was not established until the late L4 stage (data not shown). This temporal difference in the onset of *unc-4* expression is consistent with the idea that the histone methylation-associated transcriptional repression is established prior to the external EGF signal, which derepresses *unc-4* gene by presumably removing the repressive histone modification.

In fact, histone demethylases were also required for the derepression of *unc-4* in vulval VC neurons. Among the 13 genes encoding predicted histone demethylases in *C. elegans*, we found that mutations in *jmjd-2*, *jmjc-1*, and *spr-5* significantly reduced *unc-4* expression in the VC4 and VC5 cells, but not in the *unc-4*-expressing head neurons (Figure 5E and Table S2). *spr-5* encodes the *C. elegans* homolog of human LSD1. The human enzyme demethylates both H3K4me2 and H3K9me2 [43], [44], but only the H3K4me2 demethylase activity of SPR-5 has been studied in *C. elegans* [45]. If SPR-5 does demethylate H3K9me2, it may help remove the repressive histone modification on *unc-4* gene. *jmjd-2* and *jmjc-1* encode homologs of human JMJD2a and MINA proteins respectively, both of which are involved in the demethylation of H3K9me3 [46]–[48], but the functions of these *C. elegans* proteins have not been studied. Thus, the removal of the repressive H3K9me2/3 mark could activate *unc-4* expression in vulval VC neurons.

### LIN-11 acts downstream of EGFR signaling to induce *unc-4* expression

Given the fact that the TF LIN-11 is required to induce *unc-4* expression in VC4 and VC5 cells, we expected the doubles of *lin-11* with the epigenetic factors should have no *unc-4* expression at all. However, to our surprise, *lin-11* double mutants with *pqe-1*, *cec-3*, *met-2*, *hpl-2*, and *lin-13* all showed ectopic *unc-4* expression in the six VC neurons (Figure 6C). This result suggests that LIN-11 does not directly activate *unc-4* transcription. Instead, LIN-11 may be the downstream target of the EGFR signaling that helps remove the repressive chromatin modification of *unc-4* gene.

LIN-11 was also needed for the induction of ectopic *unc-4* expression in the multivulva mutants, which have excessive EGF signals from the pseudovulvae, since the *unc-4* expression in VC neurons near the pseudovulvae was prevented by mutation of *lin-11* (Figure 6B). Thus, LIN-11 is required to alleviate the epigenetic silencing of *unc-4* in VC neurons in response to the differentiation cue from vulval cells.

### Ectopic *unc-4* expression in VC neurons causes egg-laying defects

VC neurons regulate egg laying in two ways: neuromuscular synapses from VC neurons activate the vulval muscle vm2 cell and allow eggs to be laid; and extrasynaptic release of ACh as a neuromodulator from VC neurons prevents egg laying by inhibiting the HSN neurons, which promote egg laying [5]. Since UNC-4 upregulates the expression of choline acetyltransferase (CHA-1) and the synaptic vesicle ACh transporter UNC-17 post-transcriptionally, mutation of *unc-4* leads to reduced release of ACh, increased HSN activity, and thus hyperactivate egg laying [49]. Mutations in *cha-1* and *unc-17* also result in hyperactive egg laying [6], supporting the role of ACh in inhibiting HSN activity.

**Figure 7:**
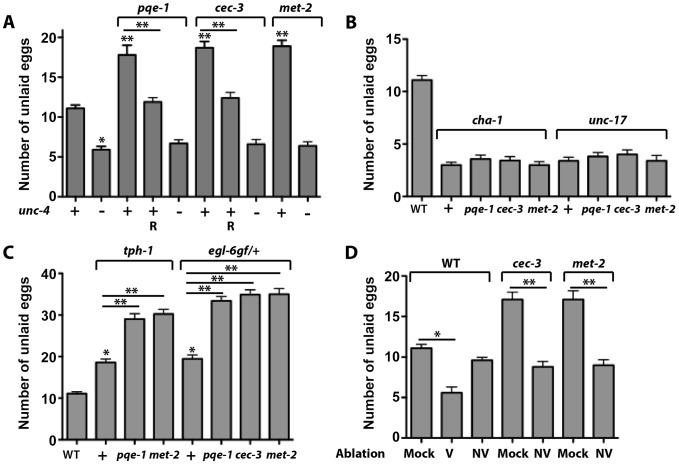
More eggs are retained by animals expressing *unc-4* in all VC neurons. (A) *pqe-1(u825)*, *cec-3(u830)*, and *met-2(n4256)* animals retain more eggs than wild type at 36 hours after becoming adults. This phenotype required *unc-4* (+, present; − absent). R indicates animals expressing the wild-type copy of the indicated gene from a VC-specific promoter. At least 30 staged adults were used for each genotype here and in subsequent panels. Statistical analyses were performed between the wild-type and the single mutants (asterisks immediately above the bars) and between the mutants and rescues (asterisks above the lines). A single asterisk indicates P<0.05 and two asterisks indicate P<0.01 in a paired t-test. (B) *cha-1(p1152)* and *unc-17(e245)* are epistatic to *pqe-1*, *cec-3*, and *met-2*. (C) *tph-1(mg280)* and *egl-6gf(n592)/+* are additive with *pqe-1*, *cec-3*, and *met-2*. (D) Fewer eggs were retained when vulval (V) but not nonvulval VC (NV) neurons were ablated in wild animals at late L4 stage and eggs in the uterus were counted 24 hrs later. Most of the added egg retentions in *cec-3* and *met-2(n4256)* mutants is eliminated when the nonvulval VC neurons are similarly ablated. N = 20 for each condition.

Since all of the VC1-3 and VC6 neurons send out processes to the vulval region, ACh produced by these cells could reach the HSN neurons even if they don't directly synapse onto HSNs. Therefore, we reasoned that mutants with ectopic *unc-4* expression in the nonvulval VC neurons could produce extra amounts of ACh, causing hypersuppression of the HSNs and reduction in egg laying. Indeed, *pqe-1*, *cec-3*, and *met-2* adults retained 7–8 more eggs than wild-type animals (*pqe-1*: 17.8±1.2; *cec-3*: 18.7±0.78; *met-2*: 18.9±0.75; wild type: 11.1±0.4; mean ± SEM; N = 30; Figure 7A). Consistent with the increased egg-retention, the age of the eggs that were laid was older in the *pqe-1* and *cec-3* animals (*pqe-1*: 48%; and *cec-3*: 62% at comma stage; N = 50) than wild type (62% at 21+ cell stage and nearly no eggs at comma stage; Figure S8). VC-specific rescue of either *pqe-1* or *cec-3* restored normal egg retention levels, suggesting that these genes act within VC neurons. Egg retention in these animals appeared to require UNC-4–mediated activation of *cha-1* and *unc-17*, since *unc-4*, *cha-1*, and *unc-17* were epistatic to *pqe-1*, *cec-3*, and *met-2*; double mutants all displayed hyperactive, instead of defective, egg laying (Figures 7A and 7B).

Consistent with the model that *unc-4* inhibits egg laying through its regulation of ACh, we found that *pqe-1* and *met-2* increased egg retention in *tph-1* mutants and *pqe-1*, *cec-3*, and *met-2* increased egg laying in heterozygotes containing one copy of an *egl-6* gain-of-function mutation (Figure 7C). The *tph-1* and *egl-6* mutations reduce egg laying and thus provide a sensitized background in which to look for egg-laying defects. *tph-1* encodes tryptophan hydroxylase, which synthesizes serotonin in HSN neurons [50]. Because serotonin is released by the HSN neurons to promote egg laying, mutation of *tph-1* leads to reduced egg-laying activity. *egl-6* encodes an FMRFamide neuropeptide receptor, the receptor of neuropeptide FLP-10 and FLP-17, which acts additively with ACh to inhibit the HSN neurons [51]. The constitutively active gain-of-function mutation of *egl-6* resulted in hypersuppression of the HSN neurons and defects in egg laying.

To further confirm that the *unc-4*-expressing non-vulval VC neurons caused the defects in egg laying, we ablated the aberrantly differentiated nonvulval VC neurons (VC1-3 and VC6) in *cec-3* and *met-2* animals to correct the phenotype. Ablated mutant animals retained the same number of eggs as ablated wild-type animals (Figure 7D). In addition, the ablated *cec-3* and *met-2* animals laid significantly fewer late-stage eggs than unablated controls (Figure S9). Ablating the vulva-flanking VC4 and VC5 neurons in the wild-type background led to hyperactive egg laying, whereas killing the other VC neurons had very little effect (Figure 7D). These data suggest that the differentiated vulval VC neurons, which normally express *unc-4*, were responsible for reducing egg laying and balancing the behavioral output of the egg-laying circuit, whereas epigenetic silencing of *unc-4* expression in the non-vulval VC neurons prevented a further inhibition of egg laying.

## Discussion

The six VC neurons are generated in L1 larvae but diversify into subtypes, adopting different morphologies and functions at the L4 stage during vulval development. At this later time the vulva-flanking VC4 and VC5 neurons differentiate further by migrating toward the vulva, extending branched processes dorsally to innervate vulval muscles, initiating *unc-4* expression, and joining the egg-laying circuit. In contrast, the other VC neurons largely maintain their original cell shape and play only a minor role in egg laying. These later cells, however, have the potential to be more like the VC4 and VC5 cells as seen in the *dig-1* mutants or mutants with multiple vulvae.

The diversification of VC neurons into subtypes requires inhibition of vulval VC differentiation in the nonvulval VC neurons, which don't receive the LIN-3/EGF developmental cue. As shown here, *unc-4* expression is a convenient marker for this differentiation. We found that *unc-4* is silenced epigenetically in nonvulval VC neurons. This epigenetic repression involves chromatin modifiers (including the histone H3K9 methyltransferase MET-2 [13] and H3K36 methyltransferase MET-1 that may indirectly promote H3K9 methylation [13], [14]), chromatin readers (including the MBT (malignant brain tumor) domain-containing protein LIN-61 [29], the HP1-like protein HPL-2 [20]), zinc finger protein LIN-13 that helps localize HPL-2 [28]), a novel chromodomain protein CEC-3, a transcriptional repressor EFL-1 [27], and a large, acid-rich protein LIN-65 with unknown function [31]. Mutation of any of these proteins led to ectopic *unc-4* expression in all six VC neurons and resulted in the loss of subtype-specificity of the expression. Because UNC-4 regulates the level of proteins needed for the synthesis and release of ACh, the failure to restrain *unc-4* expression in only VC4 and VC5 caused hyperinhibition of HSN activity and defects in egg laying. Thus, histone modification contributes to terminal neuronal differentiation by generating the correct gene expression pattern in VC cells. This control is essential for the regulation of a specific behavior, egg laying.

In addition to these epigenetic proteins, we found that the Q/P-rich domain-containing protein PQE-1 also prevents *unc-4* expression in non-vulval VC neurons. Although we do not know how this repression works, our results showed that PQE-1 acts in a similar way to the histone methyltransferases MET-1 and MET-2, the chromodomain protein CEC-3, and the HP1/HPL-2 binding partner LIN-13 in preventing transcription and protecting cells from polyQ neurodegeneration. Consistent with previous studies [9], we found that the C-terminal RNA exonuclease domain included in the b isoform of *pqe-1* gene was dispensable for PQE-1 function, indicating the N-terminal Q/P-rich domain is mainly responsible for inhibiting *unc-4* expression. Although the Q/P-rich domain is largely known to promote protein aggregation, the C-terminus of the TF TDP-43 has a Q/P-rich region that is required for its function in silencing the testis-specific gene *SP-10* [52], [53]. Therefore, we speculate that the nuclearly localized PQE-1 protein may use its Q/P-rich domain to mediate transcriptional repression. Thus, Q/P-rich proteins may be a new class of epigenetic control factors.

Previous studies showed that repressive epigenetic modifications are essential for silencing critical developmental genes and inhibiting differentiation in stem cells. ESCs deficient in PcG proteins, which promote H3K27 trimethylation and suppress transcription, derepressed neural genes, such as *Ngns*, *Pax-6*, and *Sox-1*, and were prone to differentiate [2]. Loss of the transcriptional repressor REST (RE1-silencing TF, which recruits histone modifiers and chromatin-binding proteins) or inhibition of DNA methyltransferase and histone deacetylases (both of which suppress transcription) induces aberrant differentiation and derepression of genes related to neurogenesis in ESCs and NPCs [54]–[56]. The H3K9 methyltransferase SetDB1 also contributes to the repression of genes encoding developmental regulators and to the maintenance of ESCs [57]. Consistent with these findings, we find that mutation of histone methyltransferase *met-2* that is homologous to SetDB1 and promotes H3K9 methylation abrogated the repression of terminal differentiation marker *unc-4* in undifferentiated VC neurons.

Although our results suggest that H3K9 methylation is important for the regulation of *unc-4* expression, we cannot definitively determine whether dimethylation or trimethylation is important. Unlike SetDB1, which specifically trimethylates H3K9, MET-2, the *C. elegans* homolog of SetDB1, mediates mono- and dimethylation of H3K9 [17]. Another SET domain protein, SET-25, which is homologous to the mammalian EHMT1/G9a and Suv39h1/2, is responsible for H3K9 trimethylation in early embryos [17], but its role in larvae and adults has not been examined. *set-25* mutants did not show ectopic *unc-4* expression, so either H3K9 dimethylation is responsible for *unc-4* silencing or MET-2 or another HMT promotes H3K9 trimethylation of *unc-4* DNA in adult VC cells. Our finding that the release of *unc-4* epigenetic silencing needed *jmjd-2* and *jmjc-1*, homologs of mammalian demethylases that have demethylation activities on H3K9me3 [46]–[48], argues for the second hypothesis. Among the chromatin readers that are important for *unc-4* repression, LIN-61 and HPL-2 directly binds to H3K9me2/3 [22], [29], but the ability of CEC-3 to bind to H3K9me2/3 remains to be determined. Although HPL-2 can be indirectly recruited to H3K27me3 [21], our results suggest that H3K27 trimethylation is not the likely cause of *unc-4* silencing because neither the polycomb complex components that catalyze H3K27 trimethylation nor the predicted H3K27me2/3 demethylases had any effect on *unc-4* expression.

Developmental signals can reverse H3K9 methylation-mediated gene silencing in undifferentiated neurons to allow the differentiation to proceed. In this study, we found that EGF/LIN-3 from the developing vulF cells acts through the EGFR/RAS/MAPK signaling pathway during vulval development to override the epigenetic silencing of *unc-4* in VC neurons that are close to vulF cells. The EGF signal probably failed to activate *unc-4* expression in the nonvulval VC neurons because the physical distance between these cells and the EGF source was too great. At least three events are needed for the correct timing of *unc-4* expression in VC4-5: 1) epigenetic silencing prevents *unc-4* expression in the early L4 stage (*unc-4* is expressed in early L4 cells in *cec-3* or *met-2* mutants); 2) induction involving EGF, etc. leads to *unc-4* expression in the late L4; and 3) additional factors (the presence of a negative factor or the absence of a positive factor) prevent *unc-4* expression from the L1 to the late L3 stage. Therefore, *unc-4* transcription would be on by default in all six VC cells if H3K9 methylation did not inactivate *unc-4* prior to the differentiation cue. The epigenetic inhibition is normally relieved in VC4-5 neurons by the external signal from the developing vulva to allow the derepression of *unc-4* gene and neuronal differentiation. Although the morphological differentiation of VC neurons is not controlled by the epigenetic mechanisms, our data demonstrate that H3K9 methylation helps create subtype-specific gene expression patterns during terminal neuronal diversification.

We have also found that LIM-domain transcription factor LIN-11 is required to derepress *unc-4* in the vulval VC neurons. Since *lin-11* acts similarly as the EGFR signaling genes in genetic interaction studies, LIN-11 is likely to be part of the EGFR pathway, probably the downstream target of the signaling, and helps remove the repressive histone modification. Although there is no report showing the direct involvement of LIM domain proteins in histone demethylation, a close correlation between the expression patterns of lysine-specific histone demethylase 1 (LSD1) and four and a half LIM-domain protein 2 (FHL2) was found in prostate cancer [58]. Since both FHL2 and LSD1 serve as coactivators of the androgen receptor [59], the LIM domain protein may interact with the histone demethylase to activate gene expression. Since histone demethylase SPR-5/LSD1 was also required for the *unc-4* VC expression, EGFR signaling is likely to activate LIN-11, resulting in the removal of H3K9 methylation and the derepression of *unc-4*.

The fact that vulF cells from the developing vulva send out EGF signal to induce VC neuron differentiation suggested a coordination between the formation of the epithelial vulval structure and the differentiation of the egg-laying neurons. In fact, signals from the 1° vulval cells also control the axonal outgrowth, branching and fasciculation of the HSN neurons [60], which together with VC neurons form the egg-laying circuit. Moreover, only the vulva-flanking VC neurons undergo morphological changes and express *unc-4*, and similarly the proximity of the HSN cell body to vulval cells is important for HSN axonal guidance [60], supporting the hypothesis that communication between the vulval epithelial cells and neurons depends on secreted extracellular molecules. As VC and HSN neurons are generated much earlier than the vulva, signals from the developing vulva need to activate these neurons by regulating gene expression, inducing axonal outgrowth, and eventually joining them together to form the neural circuit. Therefore, the terminal differentiation of these neurons requires highly coordinated cell-cell interactions with the epithelial tissues.

Finally, our findings are consistent with recent discoveries on the epigenetic regulation of cellular differentiation by H3K9 methylation in various tissues. Ling *et al.* found that mutation of the H3K9 methyltransferase G9a induced abnormal myogenesis during skeletal muscle differentiation by de-repressing the transcription of myogenic regulatory factor MyoD [61], and Herzog *et al.* found that the histone demethylase Kdm3a, which removes H3K9 methyl groups, is essential for the differentiation of mouse embryonic carcinoma cells into parietal endoderm-like cells in a mouse embryonal carcinoma model [62]. These studies demonstrate that H3K9 methylation silences developmental genes and prevents aberrant differentiation, and that the removal of this histone modification allows normal differentiation to proceed. Our studies extend these observations by showing that H3K9 methylation helps maintain the ground state among similar neurons by silencing key genes associated with terminal differentiation.

## Materials and Methods

### Strains

*C. elegans* strains were maintained at 15°C or 20°C as described by Brenner [63]. Temperature-sensitive strains were maintained at 15°C and transferred to 25°C for one generation before testing. Most strains were provided by the *Caenorhabditis* Genetics Center, which is funded by NIH Office of Research Infrastructure Programs (P40 OD010440). *cec-3(ok3432) II* and *pqe-1(ok1983) III* were generated by the International *C. elegans* Gene Knockout Consortium (http://www.celeganskoconsortium.omrf.org). *jmjc-1(tm3525) I*, *jmjd-2(tm2966) II*, *hpl-2(tm1489) III*, and *hpl-1(tm1624) X* were obtained from the National BioResource Project of Japan (http://www.shigen.nig.ac.jp/c.elegans/index.jsp). *cec-3(u830)* and *pqe-1* alleles (*u825*, *u829*, *u831*, *u832*, *u900*, *u901*, *u902*, and *u903*) were isolated in this study. *peIs304* and *pqe-1(ok1983); peIs304* strains were kindly provided by Dr. Yuichi Iino (University of Tokyo).

### Constructs and transgenes

The *unc-4p::MDM2::GFP* vector TU#703, which contains a 2.5 kb *unc-4* promoter and DNA encoding a truncated and mutated RING domain from human MDM2 attached to GFP [8], was injected together with pRF4 (containing a dominant roller marker) to generate *uIs45*, which was mapped onto the X chromosome. We also replaced GFP with RFP in TU#703 to create TU#1101 *unc-4p::MDM2::RFP*, which was used to generate transgene *uIs147*. To examine the *unc-4* expression pattern in various genetic backgrounds, we crossed *uIs45* into most of the mutants of interest except for these X-linked mutations, which were crossed with *uIs147*.

A VC-specific promoter, which contains a ∼500 bp *lin-11* promoter and a basal *pes-10* promoter, was subcloned into the Gateway pDONR P4-P1R from pDM4 vector. The pDM4 vector was a gift from Michael Koelle (Yale University). The coding regions of *pqe-1*, *cec-3*, *lin-45*, *let-60*, and *lin-11* genes were all cloned from wild type (N2) genomic DNA into Gateway pDONR 221. The resulted entry vectors, together with pENTR-VCprom, pENTR-unc-54-3′UTR, and destination vector pDEST-R4-R3 were used in the LR reaction to create the final VC promoter-driven expression vectors. The Gateway cloning method can be found at http://www.invitrogen.com/site/us/en/home/Products-and-Services/Applications/Cloning/Gateway-Cloning.html by Life Technologies (Grand Island, NY). These constructs were injected into corresponding mutants to form extrachromosomal arrays to test the VC-specific rescue of the mutant phenotype. The transgene *vsIs13[lin-11p::pes-10p::GFP]* was used as a VC-specific marker [6].

### Whole genome sequencing and identification of phenotype-causing mutations

After visually isolating the mutants, we outcrossed the mutant strains (*u825*, *u830*, and *u834*) with N2 at least 10 times and then subjected them to whole genome sequencing using an Illumina GAII genome analyzer [64]. We first identified the genetic variants by aligning the sequencing data to Wormbase reference sequences (version WS220) with MAQGene [65] and subtracting the background variants found in our wild-type strain. By visualizing the genomic positions of these variants, we discovered a variant-enriched region, which theoretically contained the phenotype-causing mutation because this region had the least chance to be recombined with wild-type chromosomes under constant selection. Within this ∼1 Mb region, we identified candidate mutations and performed complementation tests with known alleles to find the gene associated with the phenotype. We confirmed the results by PCR genotyping, testing knockout alleles of candidate genes, and injecting the cosmid or fosmid containing the wild-type copy of the gene into mutant animals and testing for rescue.

### Single-molecule fluorescent *in situ* hybridization and microscopy

Single-molecule fluorescence *in situ* hybridization was performed on young adult animals as described [24]. Forty-eight 20-nucleotide probes for *unc-4* mRNA were designed using the program at www.biosearchtech.com/customoligos and synthesized and coupled to Cy5 by BioSearch Technologies (Novato, CA). We imaged the animals using a Zeiss Axio Observer Z1 inverted microscope with a CoolSNAP HQ2-FW camera (Photometrics, Tucson, AZ) and appropriate filters for Cy5. We collected stacks of 20–35 images spaced 0.3 µm apart for each individual neuron and counted the number of fluorescent spots per neuron.

Other imaging was conducted on either the same Zeiss Observer Z1 microscope with the CoolSnap camera or a Zeiss Axioskop II with a SPOT-2 slider camera (SPOT Imaging Solutions, Sterling Heights, MI).

### Laser ablation

Ablations were performed as described previously [66]. Briefly, L4 larvae of strains carrying either *uIs45* or *vsIs13* as VC markers were placed in 1 µl of M9 buffer on a 2% agarose pad containing 1 mM sodium azide. GFP-positive cells were identified using a Zeiss Axioplan 2 equipped with a Micropoint Laser System (Photonic Instruments, Inc.), and their nucleoli were repeatedly targeted with the laser until they appeared ruptured. Mock-ablated animals were placed on the same pad and exposed to fluorescence excitation light for the same period of time, but not shot with the laser. The animals were recovered and examined 24–30 hours later with a fluorescence dissecting microscope to ensure absence of GFP-positive cells. Thirty hours after the ablation procedure, animals were assayed for egg-laying activity. To ablate vulF cells, *syIs66[B0034.1::pes-10::GFP]* was crossed into *uIs45* to help visualize the VulF cells, and *unc-4* expression in VC neurons was examined 24 hours after the ablation.

### Egg-laying assay

The average number of unlaid eggs in the uterus and the percentage of freshly laid eggs at various stages were quantified as described [51], [67]. We collected late L4 animals and cultured then at 20°C for 36 hr. In the unlaid egg assay, 30 synchronized adults were individually dissolved in 5% sodium hypochlorite, and their eggs were counted. In the egg-staging assay, 20 staged adults were placed on a thin lawn of OP50 bacteria and allowed to lay eggs for 1 hr. Each egg was examined under a dissecting microscope and categorized into six different stages according to previous studies (Ringstad and Horvitz, 2008). Eggs with eight cells or fewer were classified as “early stage”. Eggs at the comma stage or later stages were classified as “late stage”. Every experiment was repeated three times independently.

## Supporting Information

Figure S1 *uIs45[unc-4p::MDM2::GFP]* (A) labels fewer neurons in the ventral nerve cord than *uIs151[unc-4p::GFP]* (B). Times are hours after hatching.
(JPG)

Figure S2 Expression of *uIs45* in the ventral nerve cord at various developmental times. Scale bar = 20 µm for the first four sets of images and 50 µm for the last three sets.
(JPG)

Figure S3 *ceh-20* mutants ectopically express *unc-4* in VA neurons. (A) *uIs45* expression in a *ceh-20(u843)* adult. Arrows point to the VA neurons that abnormally express the reporter. The identity of these neurons was confirmed by the labeling of a VA marker *wdIs3[del-1p::GFP]* (data not shown). Scale bar = 100 µm. (B) The structure of *ceh-20* gene and the position of the *u843* mutation. (C) Rescue of the *unc-4* ectopic expression by injection of *ceh-20(+)* into *ceh-20(u843); uIs45* animals rescues the *unc-4* expression defect. (D) Absence of expression of VC marker *vsIs13* in *ceh-20(u843)* animals.
(JPG)

Figure S4 The extra *unc-4*-expressing neurons in (A) *pqe-1*, (B) *cec-3*, and (C) *met-2* animals are VC neurons. Cells were labeled with *uIs147[unc-4p::MDM2::RFP]* and the VC marker *vsIs13*.
(JPG)

Figure S5 *pqe-1a_prom::RFP* is expressed in the VC neurons (arrows).
(PNG)

Figure S6 *pqe-1* acts similarly to genes involved in epigenetic silencing in suppressing transcription and protecting neurodegeneration. (A) Effect of *pqe-1*, *cec-3*, *met-2*, and *lin-13* on the expression of *peIs304[lin-11pAD::Venus, tdc-1p::mRFP, rol-6(d)]* in the AIZ neurons. Otherwise wild-type animals had weak and variable expression, the mutations stabilized and enhanced *peIs304* expression. Green represents the percentage of animals with Venus expression in both AIZL and AIZR neurons; orange and red represent the percentage of animals with only one or no AIZ neuron labeled. N = 45 animals for each genotype. (B) Effect of *pqe-1*, *cec-3*, *met-2*, *met-1* and *lin-13* mutation on polyQ-induced neurodegeneration. N≥80 ASH neurons.
(JPG)

Figure S7 EGFR signaling components are required for *unc-4* expression in vulval VC neurons. (A–B) Expression of *uIs45* in *lin-3(n1058)*, *let-23(sy12)*, *let-60(n2021)*, and *lin-45(n2021)* vulvaless adults. The asterisk marks the position where the vulva should have developed. Scale bars = 100 µm. (C) Expression of *let-60(+)* and *lin-45(+)* from a VC-specific promoter rescues the *uIs45* expression in the VC4 and VC5 neurons (white arrows) of *let-60(n2021)* and *lin-45(n2018)* animals, respectively. The DIC images, however, show that these animals are still vulvaless. Scale bars = 20 µm.
(JPG)

Figure S8 Eggs laid by *pqe-1* and *cec-3* animals contain late stage embryos. Freshly laid eggs from wild type and *unc-4(e120)*, *cha-1(p1152)*, *pqe-1(u825)*, and *cec-3(u830)* mutants were categorized into different stages (according to Ringstad and Horvitz, 2008). At least one hundred eggs were used in this assay.
(JPG)

Figure S9 Nonvulval VC neurons that express *unc-4* cause egg-laying defects. Vulval (V) or nonvulval VC (NV) neurons were ablated in wild-type, *cec-3(u830)*, or *met-2(n4256)* late L4 larvae. 24 hours after the ablation, about 60 freshly laid eggs from 15 animals were examined. Eggs with eight cells or fewer were classified as “early stage”. Eggs at the comma stage or later stages were classified as “late stage”.
(JPG)

Table S1 A list of mutant alleles identified from the genetic screen looking for ectopic *unc-4* expression in adults. Strain TU3076 [*uIs45*] was used for the screen.
(DOCX)

Table S2 The penentrance of reduced *unc-4* vulval VC neurons expression phenotype in a list of mutants defective in histone demethylases. Either *uIs45* or *uIs147* was crossed into these mutants and the *unc-4* expression pattern was examined in homozygotes mutants.
(DOCX)

Text S1 Additional materials and methods about the mosaic analysis and the assessment of polyQ-induced neurodegeneration.
(DOCX)
