# Supplementary figures and images for: Histone Methylation Restrains the Expression of Subtype-Specific Genes during Terminal Neuronal Differentiation in Caenorhabditis elegans
Source: PLoS Genet. 2013 Dec 12;9(12):e1004017. doi: 10.1371/journal.pgen.1004017 (PMC3861114; doi:10.1371/journal.pgen.1004017)

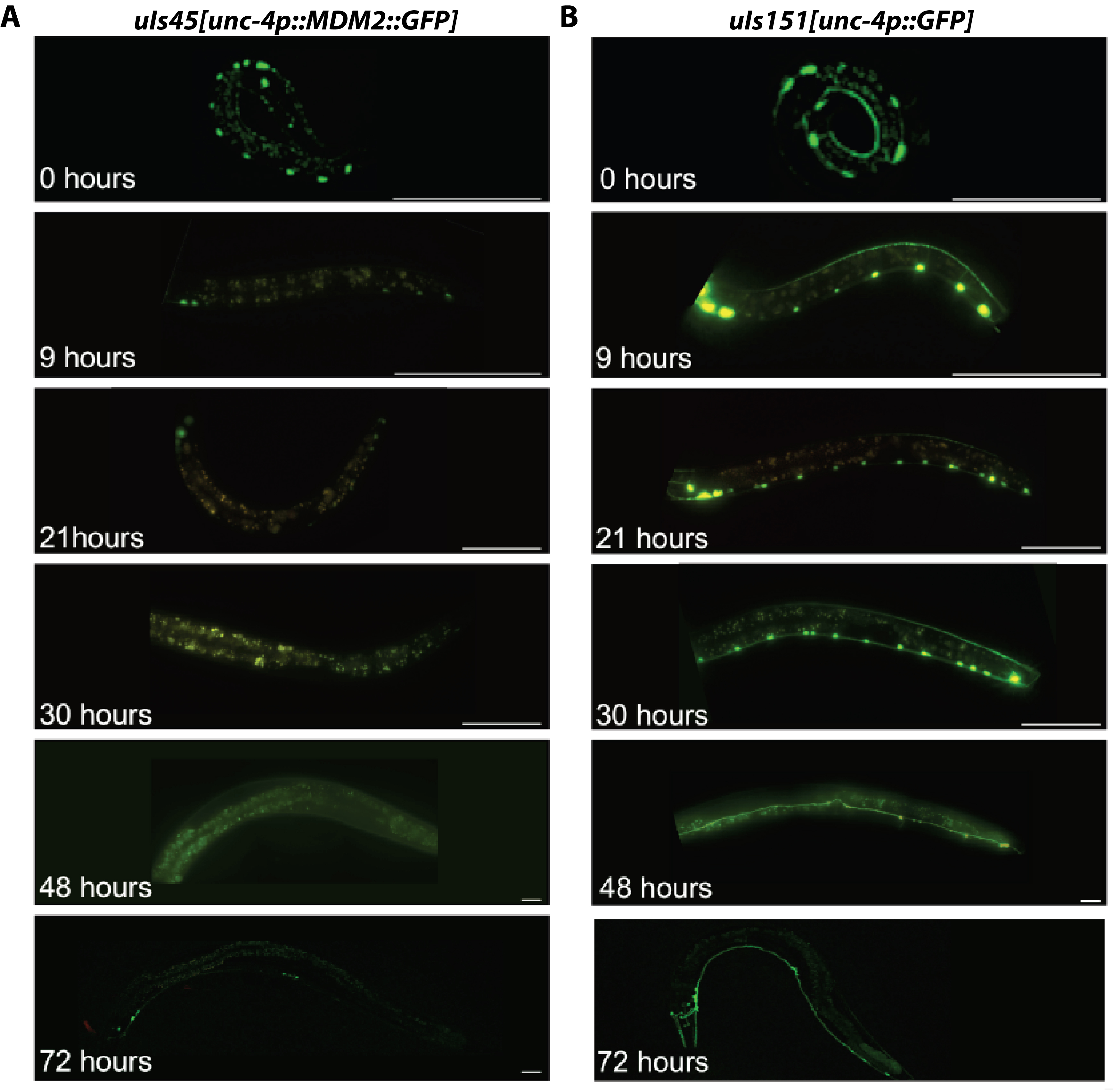

Supplement: Figure S1 — uIs45[unc-4p::MDM2::GFP] (A) labels fewer neurons in the ventral nerve cord than uIs151[unc-4p::GFP] (B). Times are hours after hatching. (JPG) [file pgen.1004017.s001.jpg]

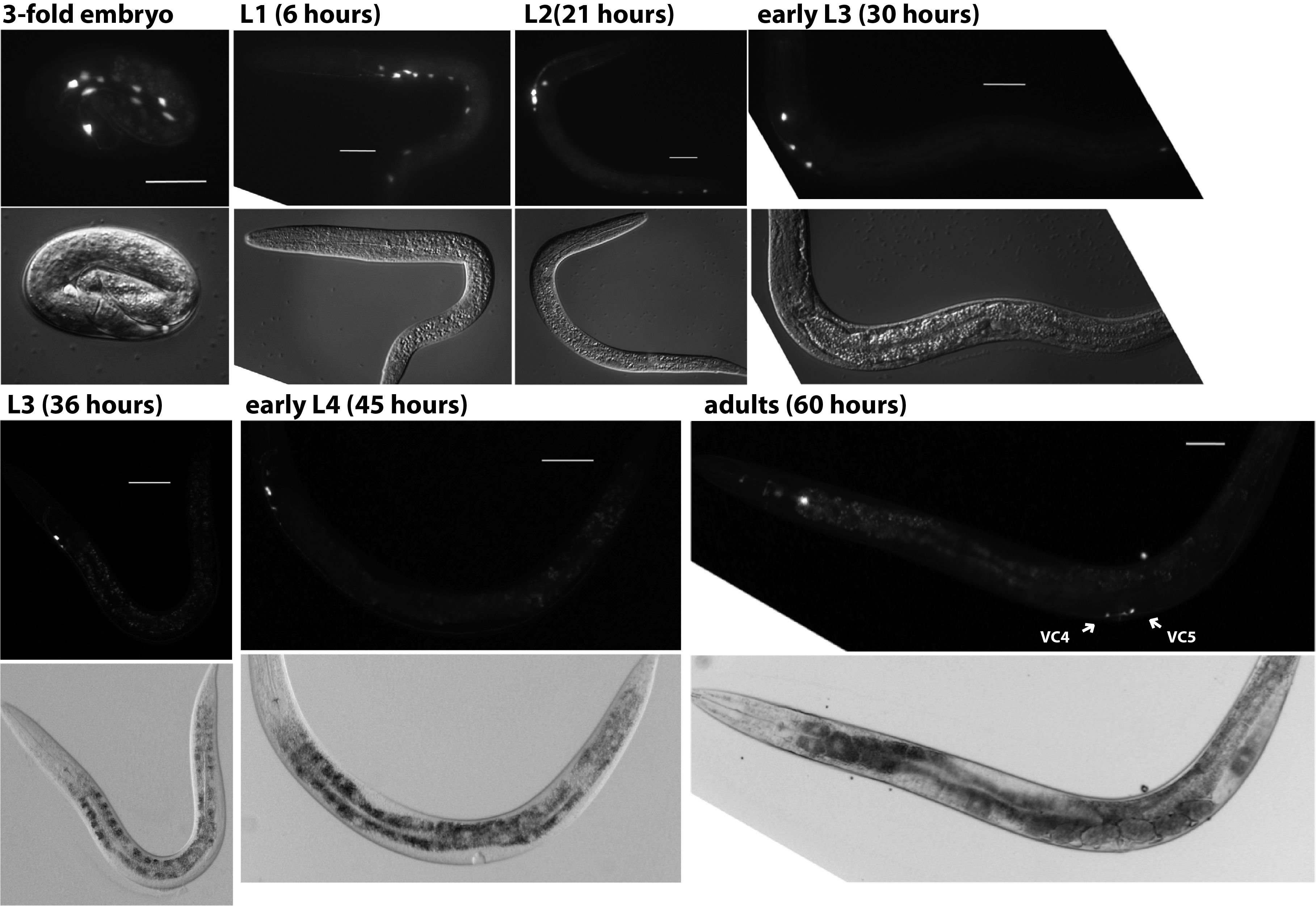

Supplement: Figure S2 — Expression of uIs45 in the ventral nerve cord at various developmental times. Scale bar = 20 µm for the first four sets of images and 50 µm for the last three sets. (JPG) [file pgen.1004017.s002.jpg]

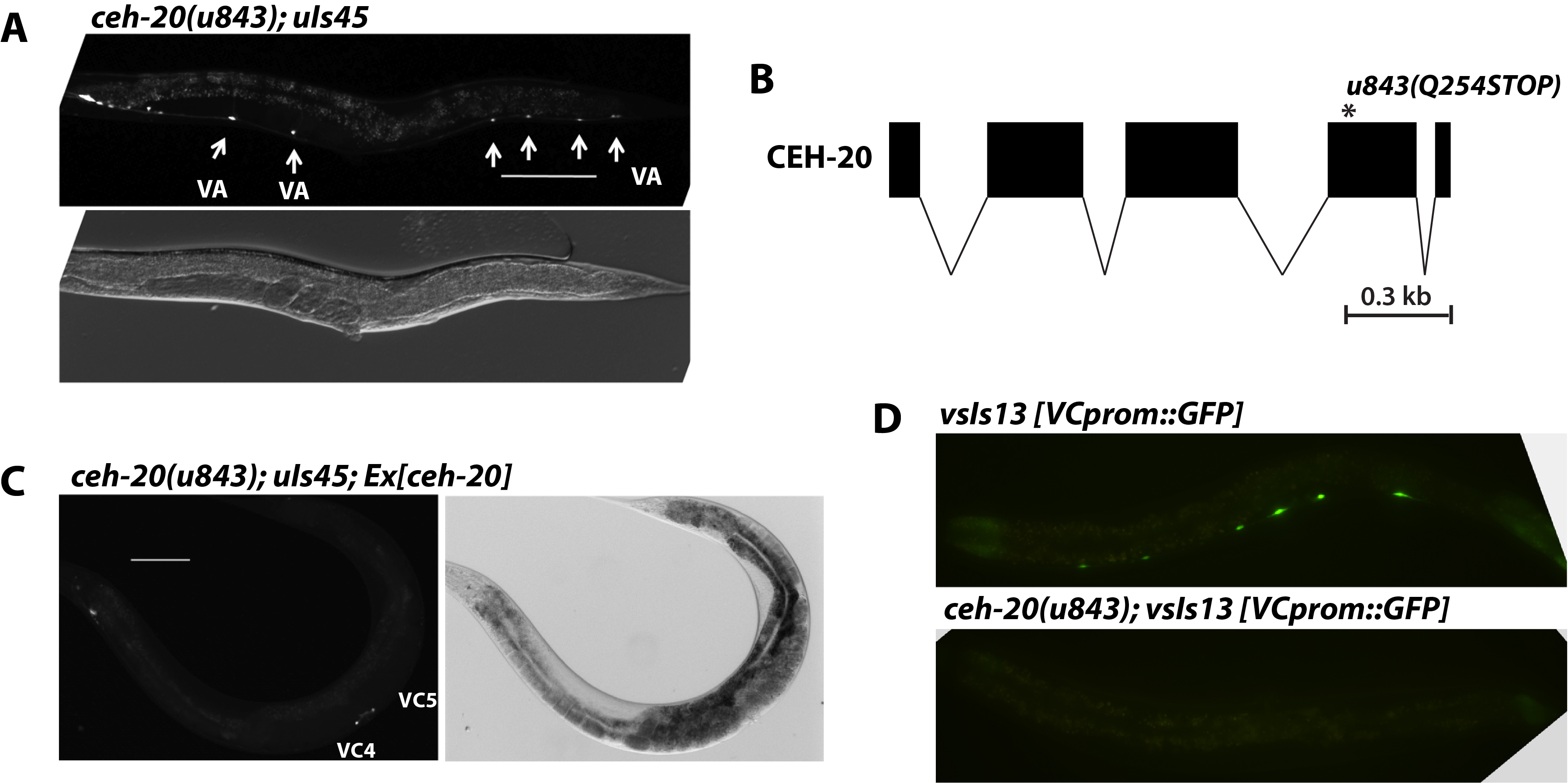

Supplement: Figure S3 — ceh-20 mutants ectopically express unc-4 in VA neurons. (A) uIs45 expression in a ceh-20(u843) adult. Arrows point to the VA neurons that abnormally express the reporter. The identity of these neurons was confirmed by the labeling of a VA marker wdIs3[del-1p::GFP] (data not shown). Scale bar = 100 µm. (B) The structure of ceh-20 gene and the position of the u843 mutation. (C) Rescue of the unc-4 ectopic expression by injection of ceh-20(+) into ceh-20(u843); uIs45 animals rescues the unc-4 expression defect. (D) Absence of expression of VC marker vsIs13 in ceh-20(u843) animals. (JPG) [file pgen.1004017.s003.jpg]

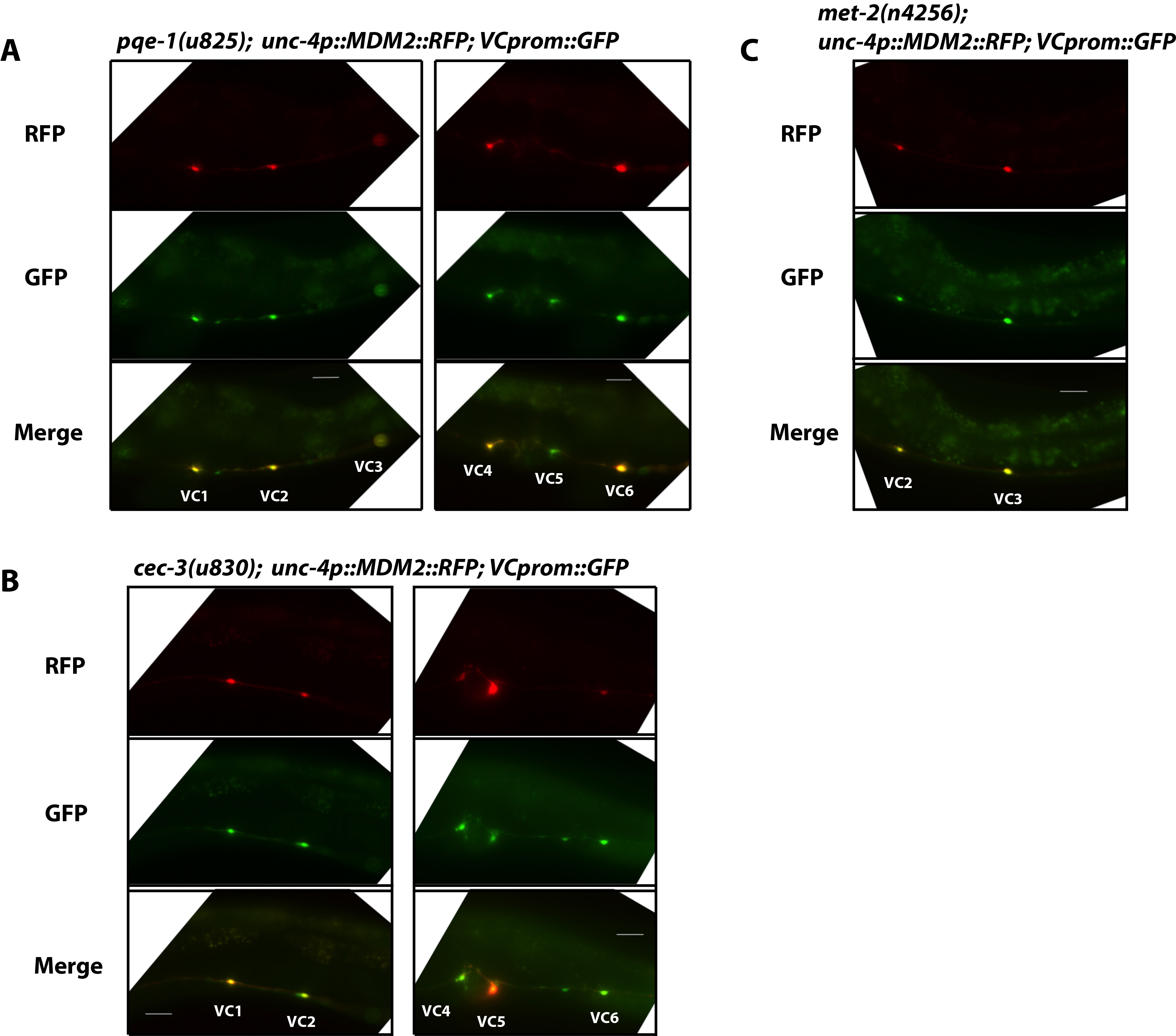

Supplement: Figure S4 — The extra unc-4-expressing neurons in (A) pqe-1, (B) cec-3, and (C) met-2 animals are VC neurons. Cells were labeled with uIs147[unc-4p::MDM2::RFP] and the VC marker vsIs13. (JPG) [file pgen.1004017.s004.jpg]

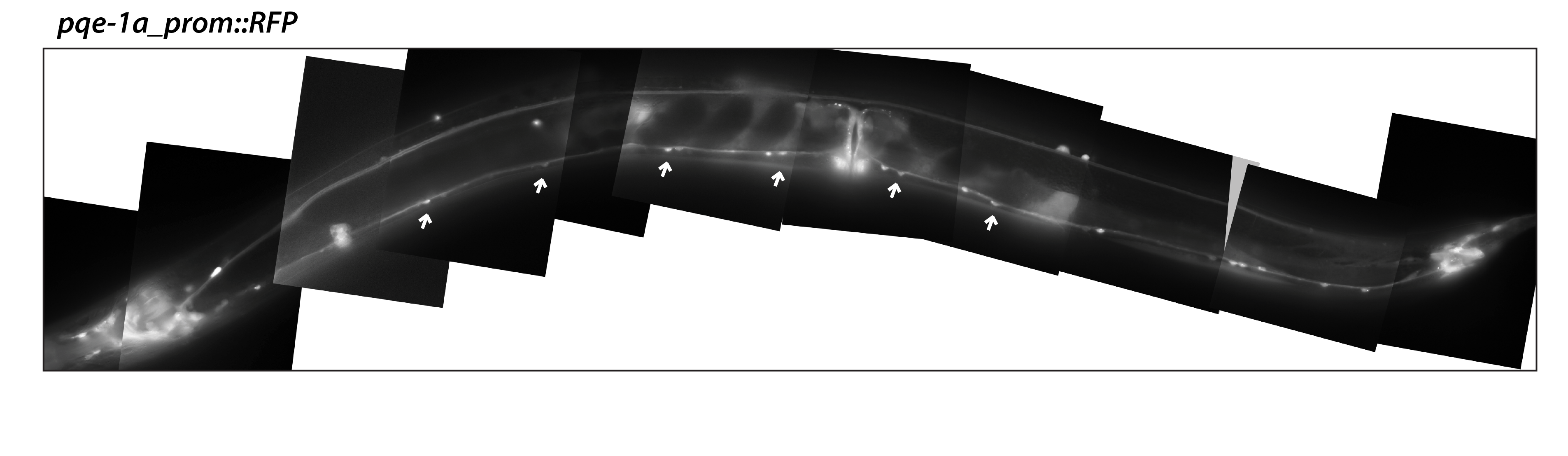

Supplement: Figure S5 — pqe-1a_prom::RFP is expressed in the VC neurons (arrows). (PNG) [file pgen.1004017.s005.png]

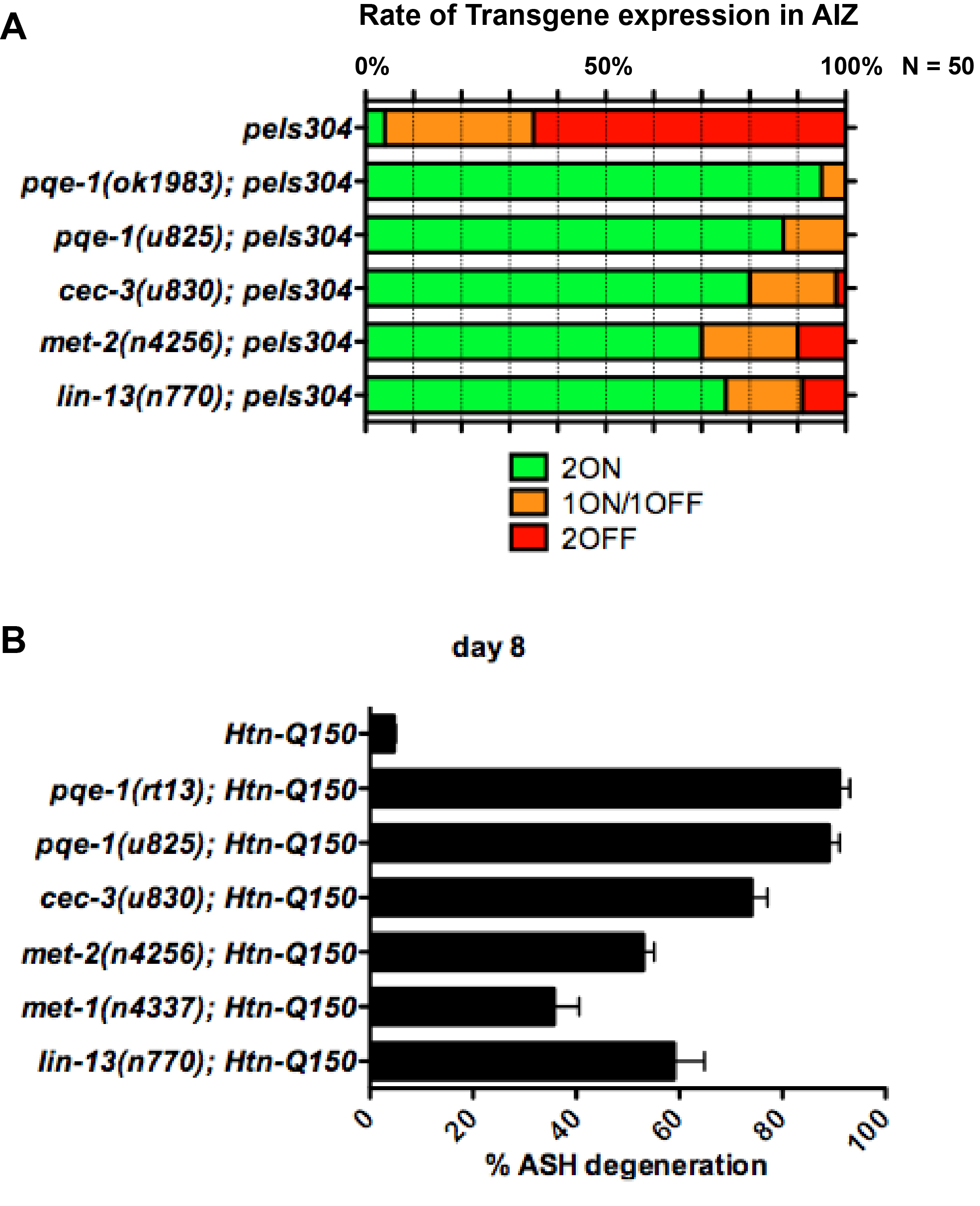

Supplement: Figure S6 — pqe-1 acts similarly to genes involved in epigenetic silencing in suppressing transcription and protecting neurodegeneration. (A) Effect of pqe-1, cec-3, met-2, and lin-13 on the expression of peIs304[lin-11pAD::Venus, tdc-1p::mRFP, rol-6(d)] in the AIZ neurons. Otherwise wild-type animals had weak and variable expression, the mutations stabilized and enhanced peIs304 expression. Green represents the percentage of animals with Venus expression in both AIZL and AIZR neurons; orange and red represent the percentage of animals with only one or no AIZ neuron labeled. N = 45 animals for each genotype. (B) Effect of pqe-1, cec-3, met-2, met-1 and lin-13 mutation on polyQ-induced neurodegeneration. N≥80 ASH neurons. (JPG) [file pgen.1004017.s006.jpg]

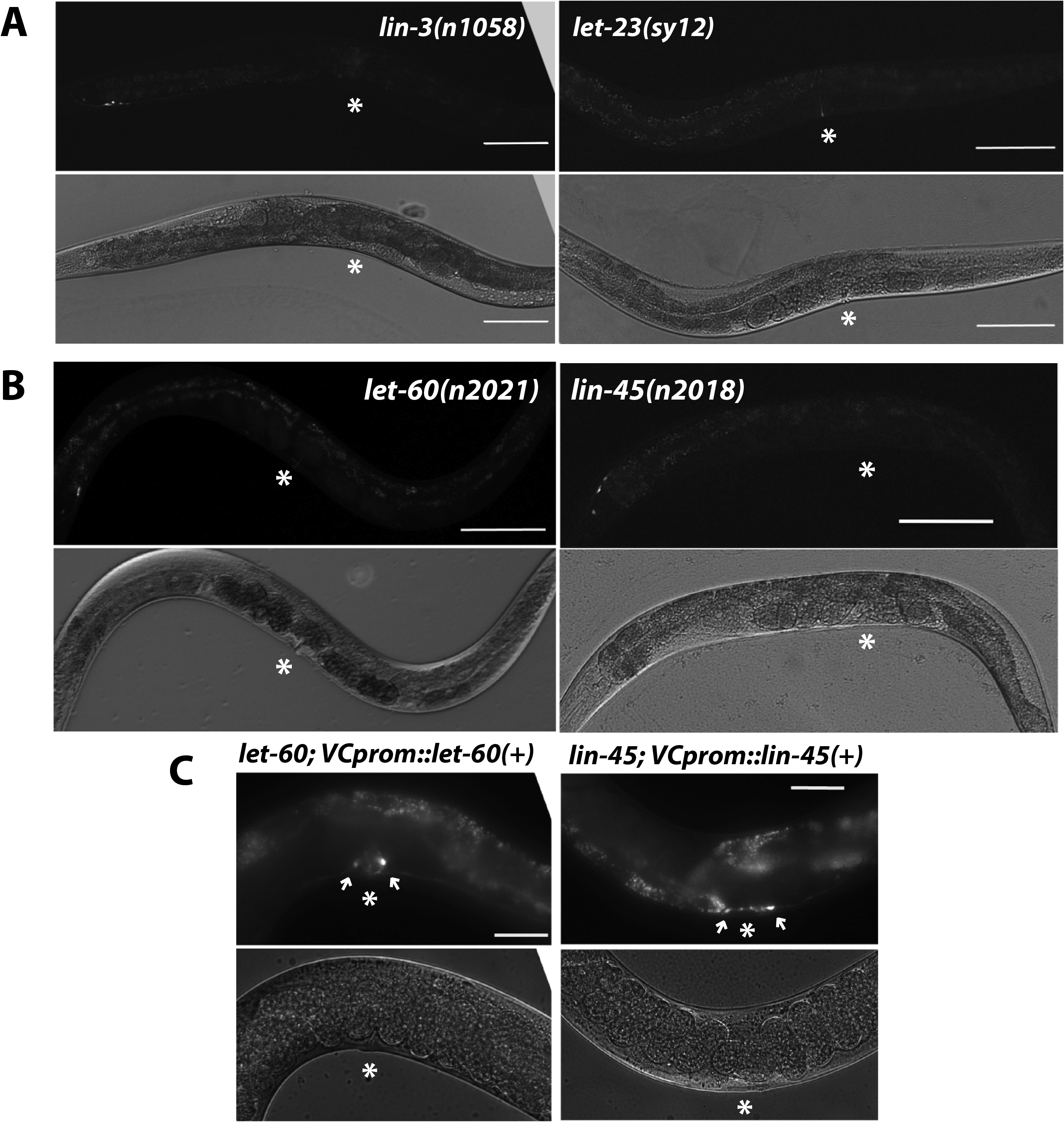

Supplement: Figure S7 — EGFR signaling components are required for unc-4 expression in vulval VC neurons. (A–B) Expression of uIs45 in lin-3(n1058), let-23(sy12), let-60(n2021), and lin-45(n2021) vulvaless adults. The asterisk marks the position where the vulva should have developed. Scale bars = 100 µm. (C) Expression of let-60(+) and lin-45(+) from a VC-specific promoter rescues the uIs45 expression in the VC4 and VC5 neurons (white arrows) of let-60(n2021) and lin-45(n2018) animals, respectively. The DIC images, however, show that these animals are still vulvaless. Scale bars = 20 µm. (JPG) [file pgen.1004017.s007.jpg]

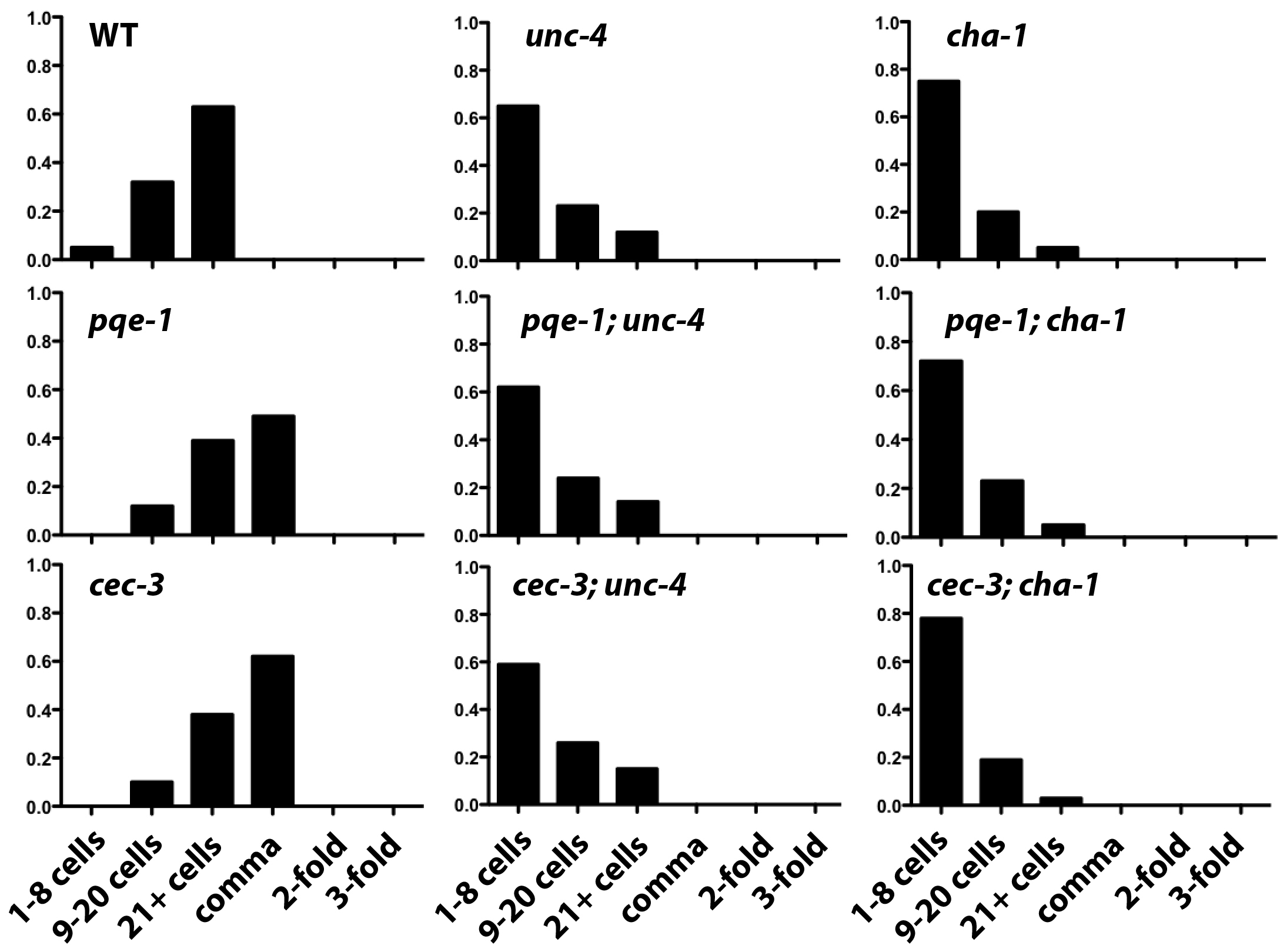

Supplement: Figure S8 — Eggs laid by pqe-1 and cec-3 animals contain late stage embryos. Freshly laid eggs from wild type and unc-4(e120), cha-1(p1152), pqe-1(u825), and cec-3(u830) mutants were categorized into different stages (according to Ringstad and Horvitz, 2008). At least one hundred eggs were used in this assay. (JPG) [file pgen.1004017.s008.jpg]

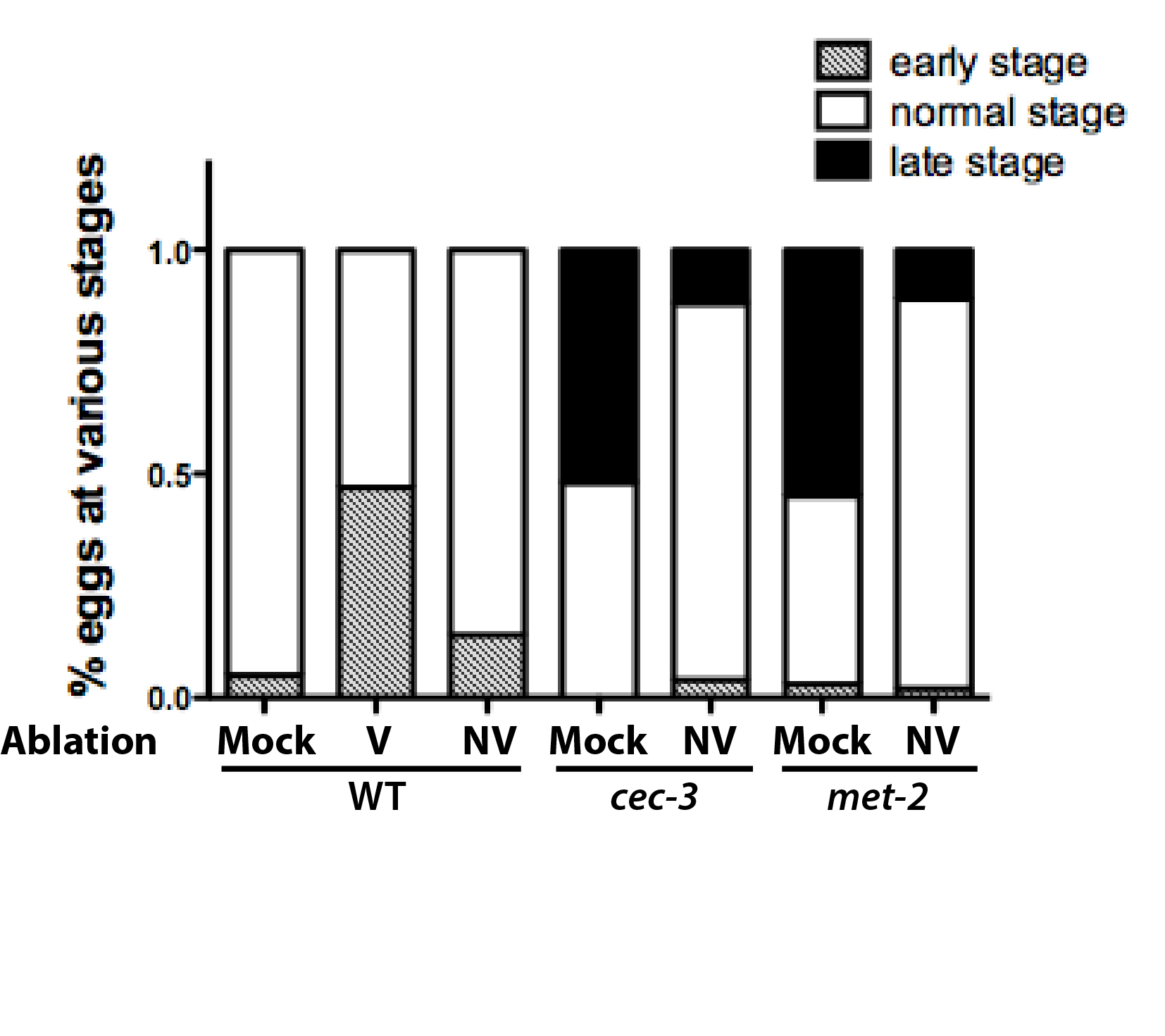

Supplement: Figure S9 — Nonvulval VC neurons that express unc-4 cause egg-laying defects. Vulval (V) or nonvulval VC (NV) neurons were ablated in wild-type, cec-3(u830), or met-2(n4256) late L4 larvae. 24 hours after the ablation, about 60 freshly laid eggs from 15 animals were examined. Eggs with eight cells or fewer were classified as “early stage”. Eggs at the comma stage or later stages were classified as “late stage”. (JPG) [file pgen.1004017.s009.jpg]
