# Supplementary material for: Histone Methylation Restrains the Expression of Subtype-Specific Genes during Terminal Neuronal Differentiation in Caenorhabditis elegans
Source: PLoS Genet. 2013 Dec 12;9(12):e1004017. doi: 10.1371/journal.pgen.1004017 (PMC3861114; doi:10.1371/journal.pgen.1004017)
Supplement: Table S1 — A list of mutant alleles identified from the genetic screen looking for ectopic unc-4 expression in adults. Strain TU3076 [uIs45] was used for the screen. (DOCX) [file pgen.1004017.s010.docx]

**Table S1.** Mutations causing abnormal *unc-4* expression patterns in adults.

| **Allele** | **Gene affected** | **Molecular Change** | **Phenotype** | **Penetrance** |
| --- | --- | --- | --- | --- |
| *u825* | *pqe-1* | Q179Stop | Strong GFP expression in six adult VC neurons | 100% |
| *u829* | *pqe-1* | Q253Stop | Strong GFP expression in six adult VC neurons | 100% |
| *u831* | *pqe-1* | Q202Stop | Strong GFP expression in six adult VC neurons | 100% |
| *u832* | *pqe-1* | Q195Stop | Strong GFP expression in six adult VC neurons | 100% |
| *u900* | *pqe-1* | Q285Stop | Strong GFP expression in six adult VC neurons | 100% |
| *u901* | *pqe-1* | Q735Stop | Strong GFP expression in six adult VC neurons | 100% |
| *u902* | *pqe-1* | Q291Stop | Strong GFP expression in six adult VC neurons | 100% |
| *u903* | *pqe-1* | Q741Stop | Strong GFP expression in six adult VC neurons | 100% |
| *u830* | *cec-3* | Q269Stop | Strong GFP expression in six adult VC neurons | 100% |
| *u834* | *cec-3* | Q269Stop | Strong GFP expression in six adult VC neurons | 100% |
| *u841* | *ceh-20* | Q254Stop | Strong GFP expression in 5-6 adult VA neurons | 95% |
| *u843* | *ceh-20* | Q254Stop | Strong GFP expression in 5-6 adult VA neurons | 95% |
| *u827* |  |  | GFP expression in 2-3 motor neurons in the posterior half of VNC | 90% |
| *u836* |  |  | Faint GFP expression in two neurons in the posterior half of VNC | 88% |
| *u833* |  |  | Faint GFP expression in one neurons in the posterior half of VNC | 22% |
| *u835* |  |  | Faint GFP expression in one neurons in the posterior half of VNC | 22% |
| *u837* |  |  | Expression in VC3 and two neurons in the posterior half of VNC | 36% |
| *u838* |  |  | Faint GFP expression in one neuron in the posterior half of VNC | 16% |
| *u839* |  |  | Faint GFP expression in one neuron in the posterior half of VNC | 12% |
| *u826* |  |  | Expression in two neurons in the anterior half of VNC | 24% |
| *u828* |  |  | Faint GFP expression in one neuron in the anterior half of VNC | 12% |
| *u840* |  |  | Very faint GFP expression in VC neurons | 28% |
| *u842* |  |  | Faint GFP expression in 1-2 neurons in the anterior half of VNC | 58% |
