# Supplementary material for: Histone Methylation Restrains the Expression of Subtype-Specific Genes during Terminal Neuronal Differentiation in Caenorhabditis elegans
Source: PLoS Genet. 2013 Dec 12;9(12):e1004017. doi: 10.1371/journal.pgen.1004017 (PMC3861114; doi:10.1371/journal.pgen.1004017)
Supplement: Table S2 — The penentrance of reduced unc-4 vulval VC neurons expression phenotype in a list of mutants defective in histone demethylases. Either uIs45 or uIs147 was crossed into these mutants and the unc-4 expression pattern was examined in homozygotes mutants. (DOCX) [file pgen.1004017.s011.docx]

**Table S2.** Effect of mutations in histone demethylases on vulval VC expression of *unc-4*.

| **Histone demethylase** | **Homology** | **Reduced or no *unc-4* expression in vulval VC neurons** |
| --- | --- | --- |
| *jmjd-1.1(hc184)* | JHDM1D (PHD and JmjC domain) | 3% |
| *jmjd-1.1(tm3980)* | JHDM1D (PHD and JmjC domain) | 0% |
| *jmjd-1.2(ok3628)* | Human PHF8 (PHD and JmjC domain) | N/A |
| *jmjd-2(tm2966)* | Human JMJD2a (H3K9me3 and H3K36me3) | 41% |
| *jmjd-3.1(gk387)* | Human JMJD3, UTX, UTY (H3K27me3) | 0% |
| *jmjd-3.1(gk384)* | Human JMJD3, UTX, UTY (H3K27me3) | 0% |
| *jmjd-3.2(tm3121)* | Human JMJD3, UTX, UTY (H3K27me3/me2) | 0% |
| *jmjd-3.3(tm3197)* | Human JMJD3, UTX, UTY (H3K27me3/me2) | 0% |
| *jmjd-4(tm965)* | Human JMJD4 (JmjC domain) | 0% |
| *jmjd-5(tm3735)* | Human KDM8 (JmjC domain) | 0% |
| *jmjc-1(tm3525)* | Human MINA (H3K9me3) | 44% |
| *spr-5(by134)* | Human LSD1 (H3K4me2 and H3K9me2) | 52% |
| *rbr-2(tm1231)* | Human KDM5B (H3K4me3/me2) | 25%* |
| *jhdm-1(ok2364)* | JmjC domain | 0% |
| *utx-1(ok3553)* | Human UTX (H3K27me3/me2) | 0% |

“N/A” indicates embryonic lethality. Strains in the list were obtained from either *Caenorhabditis* Genetics Center or the National BioResource Project of Japan.

* The results of *rbr-2(tm1231)* may be explained by its functions in vulval development (Christensen et al., 2007), since the primary vulval cells, which activate *unc-4* expression, may be defective or not generated in *rbr-2* mutants.
