# Supplementary material for: Histone Methylation Restrains the Expression of Subtype-Specific Genes during Terminal Neuronal Differentiation in Caenorhabditis elegans
Source: PLoS Genet. 2013 Dec 12;9(12):e1004017. doi: 10.1371/journal.pgen.1004017 (PMC3861114; doi:10.1371/journal.pgen.1004017)
Supplement: Text S1 — Additional materials and methods about the mosaic analysis and the assessment of polyQ-induced neurodegeneration. (DOCX) [file pgen.1004017.s012.docx]

**Supplemental experimental procedures**

**Mosaic analysis**

The *pqe-1(+)* and *cec-3(+)* fosmids together with a VC marker *lin-11p::pes-10::RFP* were injected into *pqe-1(u825); uIs45* and *cec-3(u830); uIs45*, respectively, to form extrachromosomal arrays. Stably transformed animals were examined for *uIs45* expression in VC cells that lost the rescuing array. The loss of the array was determined by the absence of the VC marker. We examined 15 mosaic animals that lost the RFP marker in at least one VC cells and checked for *unc-4* promoter-driven GFP expression in these *pqe-1(-)* or *cec-3(-)* VC cells.

**Assessment of polyQ-induced neurodegeneration**

The N terminus of human huntingtin carrying a polyQ tract consisting of 150 glutamines (Htn-Q150) was expressed from *rtIs11[osm-10p::Htn-Q150; osm-10p::GFP]* transgene to induce degeneration in ASH neurons (Faber et al. 2002). Neurodegeneration was indicated by both cell death and dye-filling defects. ASH neurons were counted as degenerated neurons if they lose the GFP expression or were unable to uptake the dye DiD at the eighth day after hatching. The percentage of ASH degeneration in *pqe-1*, *cec-3*, *met-2*, *met-1*, and *lin-13* mutants, as well as the wild-type animals, was calculated.
